# A phenomenological cartography of misophonia and other forms of sound intolerance

**DOI:** 10.1016/j.isci.2023.106299

**Published:** 2023-02-28

**Authors:** Nora Andermane, Mathilde Bauer, Ediz Sohoglu, Julia Simner, Jamie Ward

**Affiliations:** 1 School of Psychology, University of Sussex, Brighton, UK

**Keywords:** Health sciences, Behavioral neuroscience, Cognitive neuroscience

## Abstract

People with misophonia have strong aversive reactions to specific “trigger” sounds. Here we challenge this key idea of specificity. Machine learning was used to identify a misophonic profile from a multivariate sound-response pattern. Misophonia could be classified from most sounds (traditional triggers and non-triggers) and, moreover, cross-classification showed that the profile was largely transferable across sounds (rather than idiosyncratic for each sound). By splitting our participants in other ways, we were able to show—using the same approach—a differential diagnostic profile factoring in potential co-morbidities (autism, hyperacusis, ASMR). The broad autism phenotype was classified via aversions to repetitive sounds rather than the eating sounds most easily classified in misophonia. Within misophonia, the presence of hyperacusis and sound-induced pain had widespread effects across all sounds. Overall, we show that misophonia is characterized by a distinctive reaction to most sounds that ultimately becomes most noticeable for a sub-set of those sounds.

## Highlights

• Misophonia can be identified by applying machine learning to a sound-response profile
• Misophonia is linked to broad sound intolerances beyond specific triggers
• Sound intolerances linked to autism and hyperacusis are different from misophonia
• Our findings do not support a link between misophonia and ASMR

## Graphical abstract

**Figure:**
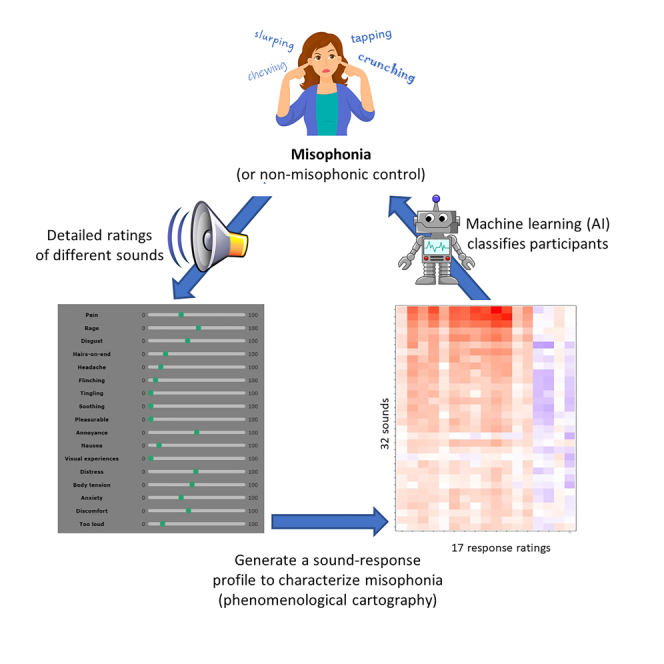

## Introduction

Misophonia is an extreme aversive reaction to certain sounds (triggers) that can substantially disrupt one’s work and social life, as well as negatively impacting on wellbeing.^1^ This research addresses the question: what is it that makes a sound misophonic? Is it something to do with the manner (oral v. non-oral) or agent of production (human v. non-human)? Or is it related to some psychoacoustic property of a sound linked to unpleasantness judgments more generally?^2^ To what extent are sounds that frequently appear as misophonic triggers also the same sounds that other people (non-misophonics) dislike? Alternatively, it may not be the sounds themselves that best identify misophonia but, instead, a particular kind of response (e.g., rage, anxiety). In this study, we introduce a novel approach that we label “phenomenological cartography” because we seek to map out what it is that makes a person misophonic using first-person (phenomenological) responses to sounds. Specifically, our map consists of 32 sounds (a mixture of typically described triggers and non-triggers) from which participants give response ratings to 17 descriptors (rage, anxiety, disgust, soothing, and so forth). In effect, determining who is misophonic and who is not (from this 17x32 array of numbers) lends itself to machine learning techniques as a form of pattern recognition (where “pattern” is synonymous with “multivariate” in statistical terms). Using this approach, we address the questions posed above by determining whether some sounds permit accurate classification of misophonia while others do not, and whether classification depends on the presence of some responses (e.g., rage) more than others. It also leaves open the possibility that there is not a “one-size-fits-all” solution. For example, misophonics may react with “rage” to the sound of chewing but “hairs on end” to creaking. This idiosyncratic sound-response pattern is discoverable by our approach.

Misophonic triggers can sometimes appear to be highly specific—such as the sound of one particular member of the family eating—and they can vary from one person to the next. On the face of it, this does not bode well for finding a generalizable misophonic sound-response pattern. But the observation that misophonic triggers are generally described as falling into certain categories of sounds, namely non-vocal oral/nasal sounds (eating, heavy breathing) or other human-made sounds (e.g. tapping) contradicts the idea they are entirely idiosyncratic.^3^ Specificity in misophonia could also be conceptualized as a minority of aversive trigger sounds against a backdrop of normal sound tolerance. Evidence in favor of this position comes from neuroimaging studies that show group differences only when misophonics, compared to non-misophonics, listen to trigger sounds (e.g., in the insular cortex) but not for other kinds of sounds.^4^^,^^5^ However, an absence of a group difference for non-trigger sounds should be interpreted with caution (limited sample sizes, limited sound exemplars). When given sounds to rate for unpleasantness or discomfort, Hansen et al.^6^ found that misophonics tended to give higher ratings for all categories of sound (human oral/nasal, human other, non-human) including for ones not traditionally described as triggers (e.g., animal sounds). In summary, there is good evidence that certain sounds are more affected by misophonia than others but the extent to which this reflects a narrow or broad pattern of sound intolerance is unclear.

Outside of misophonia itself, there is also a debate as to how to differentially diagnose different types of atypical sound sensitivity. Williams, He, Cascio, and Woynaroski^7^ argued that different forms of sound intolerance should have different kinds of specific reactions not well captured by umbrella terms like “unpleasantness.” Specifically, they argue that misophonia should elicit anger, extreme annoyance, and disgust; hyperacusis should be linked to loudness and pain; and phonophobia should be linked to fear and anxiety. They argue that decreased sound tolerance linked to the autism spectrum should have features of all three of these (i.e., be much broader in response categories). Others have argued that misophonia is primarily characterized by physical reactions such as muscle tension.^8^ Finally, a non-clinical trait linked to sound over-responsivity is ASMR, autonomous sensory meridian response^9^ for which a more accurate term may be audiovisual elicited somatosensation.^10^ ASMR is typically described as a pleasurable experience triggered by sounds linked to physical reactions such as tingling (notably to the hair/head). ASMR-triggering sounds are often described as whispering, popping, and rustling sounds. It has even been suggested that ASMR and misophonia may tend to co-occur.^11^^,^^12^^,^^13^ That is, misophonia may be linked to atypically intense experiences with sounds that, at least in some instances, have a positive valence.^8^

In the present pre-registered study, we aim to document the sound-response profile of misophonia using phenomenological cartography. We use machine learning to classify each and every sound and also perform cross-classification: to determine whether a misophonic response to, e.g., the sound of apple crunch can be used to predict misophonia status from responses to a completely different sound. Successful cross-classification would indicate a general response profile common for all sounds. Natural sounds are selected from the same three categories as Hansen et al.,^6^ i.e., including sounds not normally considered as triggers, plus a fourth category which is made up of “scrambled” versions of misophonic triggers (such that they have similar acoustic properties but are unrecognizable). As this study is part of a wider project,^14^ we are also in the fortunate position of having a set of detailed measures on other relevant individual differences that speak to the question of a differential diagnosis. For the misophonics, they were asked (in a separate session) about hyperacusis and about pain responses to sounds. Hyperacusis is typically defined by the perception that sounds feel subjectively too loud, sometimes causing pain in the ears or head.^15^^,^^16^ For the non-misophonics, we split the data according to being high/low in autistic traits,^17^ high/low sensory sensitivity across all senses,^18^ and high/low interoceptive awareness.^19^ Our goal here is to ascertain whether any misophonia profile is specific to misophonia, rather than reflecting a co-morbid trait also linked to atypical sound tolerances. Finally, in a separate study, we recruit a group of people with ASMR and add a set of ASMR trigger sounds that are presented alongside the same set of sounds given to misophonics.

## Results

The method and analysis plan was pre-registered (https://osf.io/27fgv/) and any deviations from this are explicitly noted.

### A phenomenological cartography of misophonia

**Figure 1:**
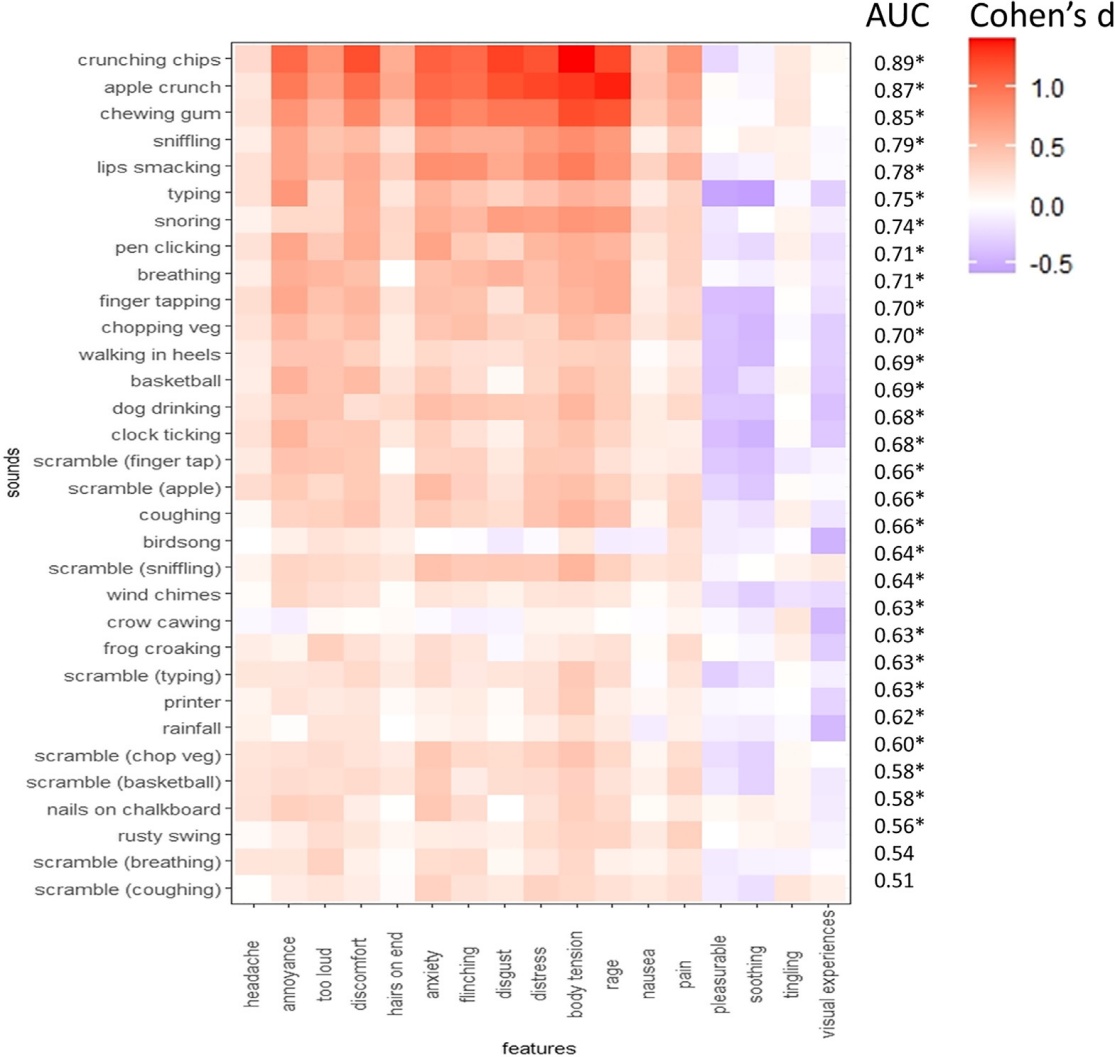
A heatmap depicting misophonic > control effect sizes (Cohen’s d) for 32 sounds and 17 response features The sounds are ranked according to the ability of a machine learning classifier to predict group membership (AUC). ∗p < 0.05 significance relative to chance classification. See also Figure S2.

For each sound, responses are measured using a 0–100 visual analog scale to descriptors such as rage and anxiety. Figure 1 shows the 32 sounds x 17 responses displayed as a heatmap, with color denoting the magnitude of the group difference for misophonics versus non-misophonics (Cohen’s d). A classifier (a machine learning algorithm) was developed for each of the 32 sounds in which it attempts to predict group status from the 17 responses, taking different training versus test resamples of the data. AUC accuracy (area-under-curve, where 0.5 is chance and 1 is perfect classification) is indicated for each row, reflecting the ability of the classifier to predict group status. For visualization purposes, the sounds are ranked, top to bottom, in terms of AUC, and the responses are organized so that frequently co-occurring ones are nearby (hierarchical clustering based on Pearson’s correlation; see Figure S6 for the corresponding dendrogram).

Thirty sounds could discriminate misophonics from controls above chance (where significance above chance was calculated via randomly permuted group labels), and two were at chance (scrambled breathing and scrambled coughing). All 30 significant results are retained after FDR correction for multiple comparisons (p < 0.05). The most discriminating sounds were well-documented misophonic triggers (crunching chips, apple crunch). However, the 32 sounds do not cluster, in a strict way, according to our initial four categories (human oral/nasal, human actions, non-human, scrambled). Instead, the overall finding is of a broad pattern of atypical responsiveness to most sounds including to some of the unrecognizable scrambled sounds. Training a single classifier across the whole 32x17 (N = 544) multivariate pattern results in a diagnostic accuracy for misophonia of AUC = 0.925 (specificity = 0.865, sensitivity = 0.802) which compares favorably against questionnaire approaches.^20^^,^^21^

With regards to which features are important for classification, the VSURF algorithm Variable Selection using Random Forests,^22^ determines the most important features for the successful performance of the classifier. Summing across all 32 classifiers the most important features were (in descending order): body tension (20), annoyance (19), anxiety (16), visual experiences (16), rage (15), discomfort (15), too loud (14), soothing (13), pleasurable (10), flinching (10), distress (9), disgust (8), pain (4), tingling (2), hairs-on-end (1), and nausea (1). Three of these features tended to yield higher responses in controls relative to misophonics (pleasurable, soothing, visual experiences)—as depicted in blue. A simple averaging of response effect sizes, down each column (ignoring sign), produces a similar rank order: body tension, rage, anxiety, distress, annoyance, discomfort, flinching, too loud, disgust, pain, soothing, hairs on end, visual experiences, pleasurable, headache, nausea, tingling.

### Comparisons between sounds: Cross-classification

**Figure 2:**
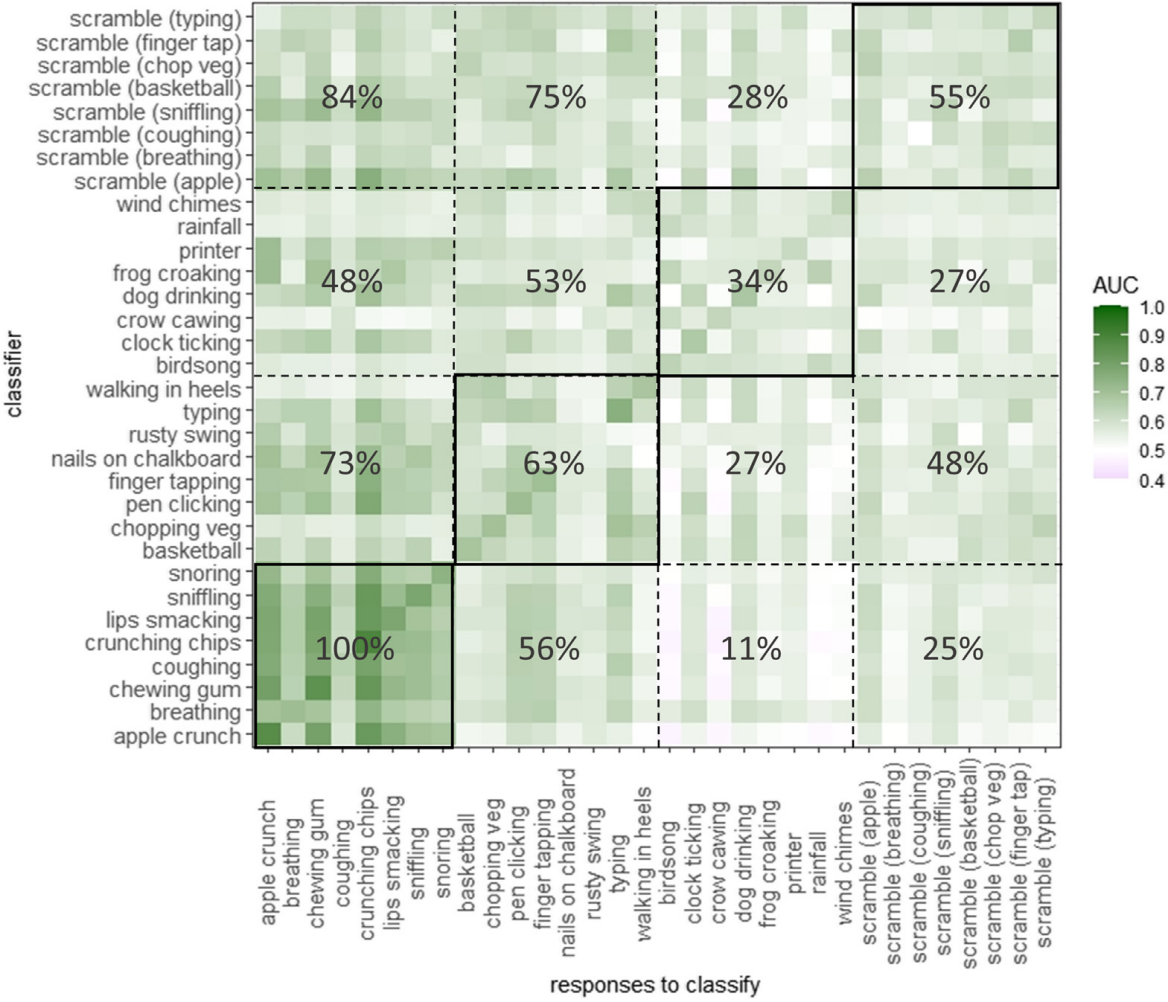
A heatmap depicting cross-classification accuracy (AUC) when a classifer trained on responses to one sound is used to predict group membership from a pattern of responses to another sound (note that the on-diagonal values are not cross-classifications) The grid lines show divisions between the four sound categories (human oral/nasal, human actions, non-human, and scrambled). The percentages within each grid cell refer to the number of cross-classifications that achieved significance (at p < 0.05 FDR corrected).

Cross-classification is a way of testing if there is a common response profile across sounds. That is, we take the classifier for one sound (e.g., the “apple crunch” classifier) and apply it to the set of responses evoked by every other sound (the ratings of rage, annoyance, and so on elicited by sounds of “chewing gum,” “dog drinking,” and so forth). We then repeated this for each of the 32 sounds in our stimulus set, yielding a 32x32 matrix of cross-classification accuracies. Figure 2 visualizes this matrix, where the on-diagonal cells represent classification performance within sounds (i.e. the AUC values already noted in Figure 1) and the off-diagonal cells represent cross-classification between sounds (again expressed as AUC). For visualization, we group the 32 sounds into the four original categories. The rows are different classifiers and the columns are different response profiles to enter into the classifier. Overall, there is a high degree of cross-classification: 58% of cross-classifications are significant at p < 0.05 and 50.1% are retained after FDR correction for multiple comparisons. Responses to human oral/nasal sounds are the easiest to classify (shown here in the left eight columns). But this is generally true across most classifiers. For example, when we train a classifier to predict misophonia status from scrambled breathing sounds it has AUC = 0.541 but when we test the same classifier on a new set of responses to chewing gum it has AUC = 0.646. That is, the response pattern from this meaningless sound acts as a good predictor (/a better predictor) of a trigger sound. The fact that the classification accuracy appears to go up in this example is because the group differences are more extreme for chewing gum. But the fact that cross-classification occurs at all is indicative of a common response pattern across a wide range of sounds.

### Comparisons between sounds: Why are some sounds rated highly?

**Figure 3:**
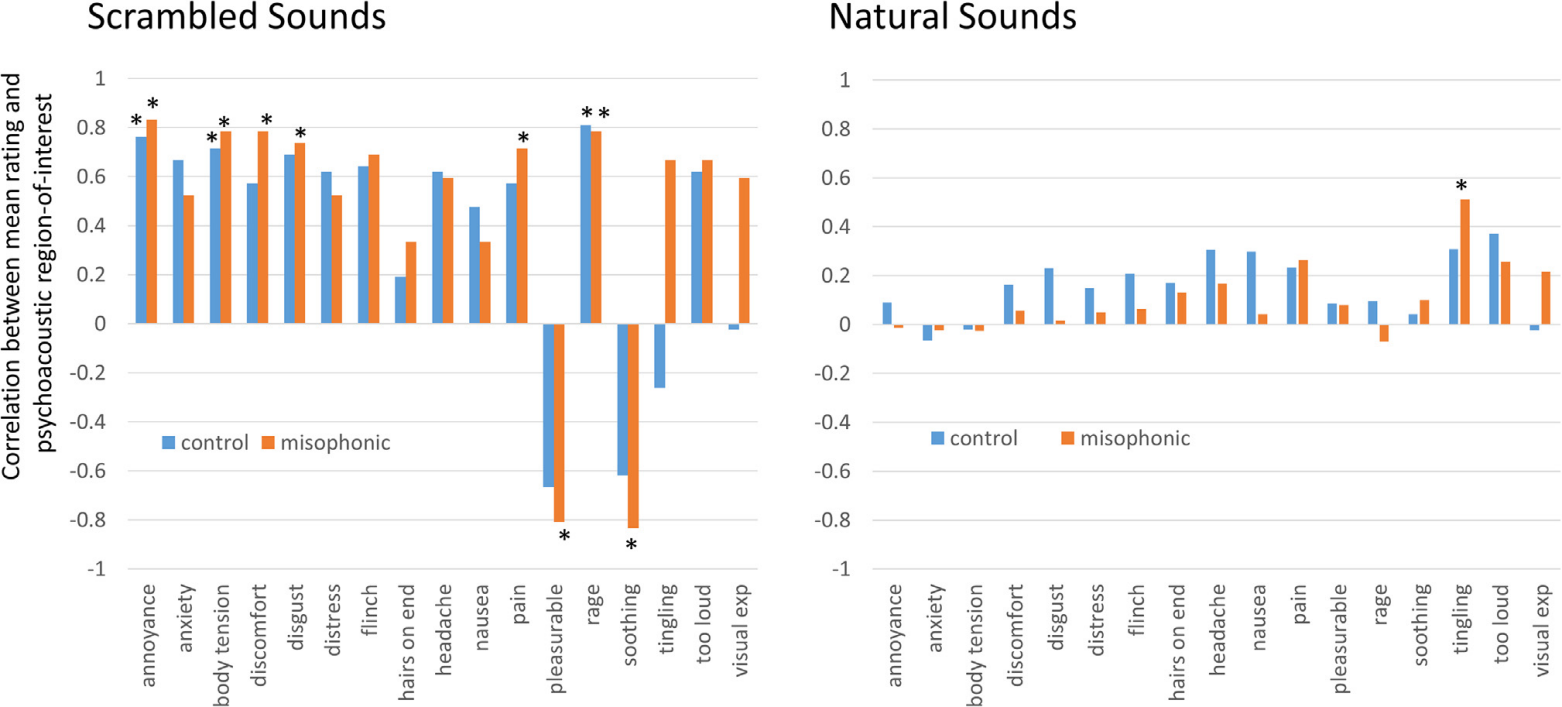
Top: Spearman’s correlations between the psychoacoustic region of interest identified by Kumar et al.^2^ and phenomenological ratings to the same sounds by misophonics and controls considering scrambled and real sounds (left and right) Note, the critical values for alpha = 0.05 are 0.643 (n = 8, i.e. scrambled sounds) and 0.344 (n = 24, i.e. natural sounds). ∗p < 0.05.

One possibility for why some sounds generate consistently high ratings across descriptors is that they contain common acoustic features. To test this, for each of our 32 sounds, we measured the energy in the psychoacoustic space of 2500–5500, Hz and 1–16 Hz temporal modulations. This has previously been shown to predict unpleasantness ratings for natural sounds.^2^ Across our set of 32 sounds, we correlated these values with the mean ratings across participants (separately for each participant group and for distress, rage, and so forth). There were four significant correlations when considering all 32 sounds together although none survived FDR correction (headache/controls r = 0.345, tingling/misophonia r = 0.471, too loud/controls r = 0.471, visual experiences/misophonia r = 0.373). However, we suggest it is not meaningful to group all the sounds together. Post hoc we noted many large (and significant) correlations for our unrecognizable scrambled sounds, but far smaller correlations for the natural sounds. This was true both for the misophonics and controls (see Figure 3). Given the limited number of sounds in our study, we conducted a reanalysis of the N = 125 natural sounds from Hansen et al.^6^ Here we use their published mean ratings of discomfort (which is also one of the 17 responses in our study) and conducted our own psychoacoustic analysis of their sound files (up to the first 30s). There was no significant correlation between mean discomfort ratings from either misophonics (r = −0.028) or controls (r = 0.004) and the energy in the region of interest (2500–5500 Hz and 1–16 Hz temporal modulations). In summary, we conclude that whilst this psychoacoustic feature has some relevance for aversive responses to sounds (as indicated by the large correlations for scrambled stimuli), it tends to be masked by other factors when sounds are easily recognizable. This occurs irrespective of misophonia status (although further research is warranted given the small number of scrambled sounds used here).

**Figure 4:**
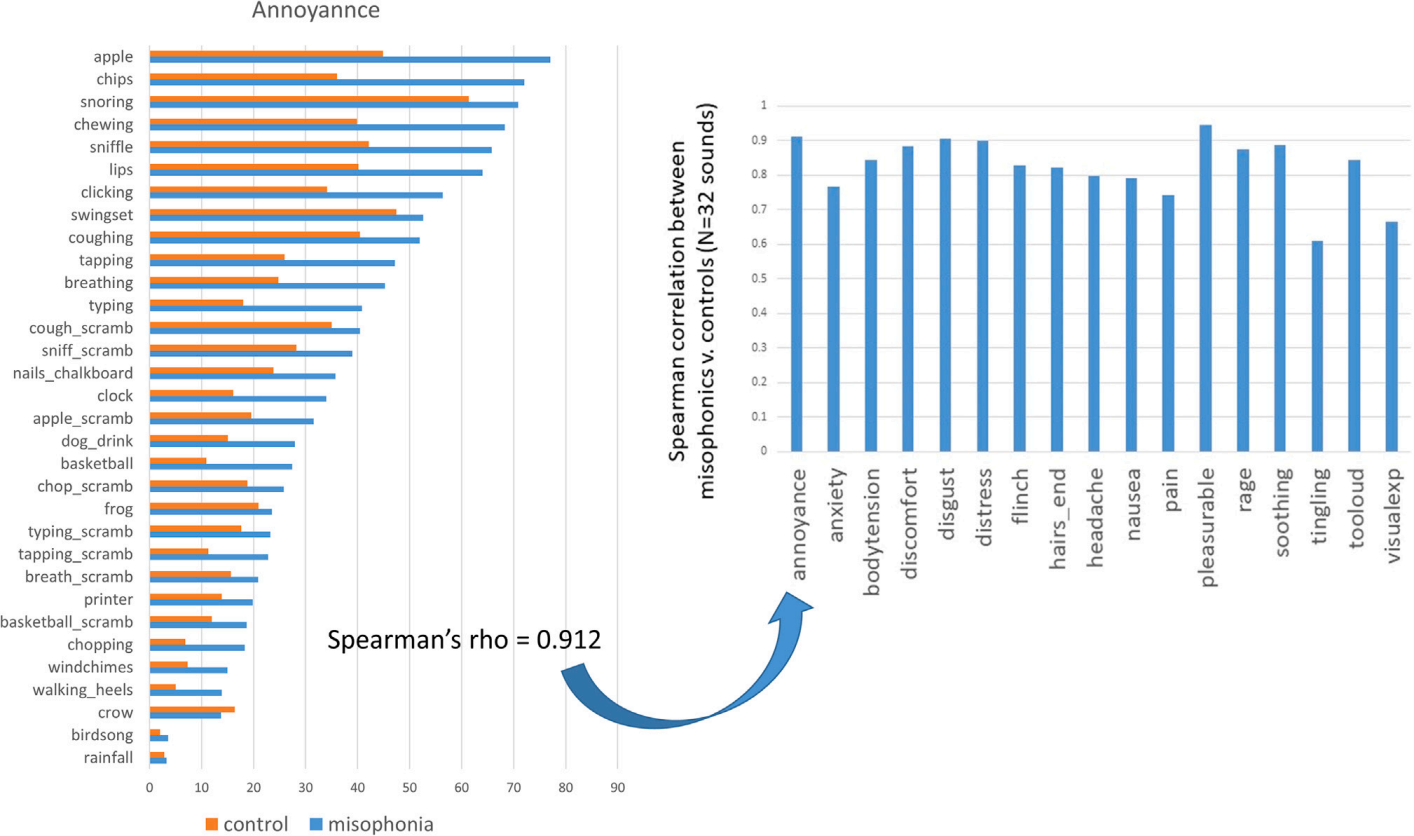
As an example, mean ratings for “annoyance” ranked from most to least annoying (according to the misophonic mean rating), with the mean correlation between ranks being 0.912 Equivalent plots for all 17 descriptors are provided in Figures S8–S12. The right panel shows Spearman’s correlations between ratings to the same sounds across groups. The critical value for alpha = 0.05 is rho ≥ 0.296 (for N = 32 sounds).

More generally, one can ask the question whether the sounds which induce annoyance (rage, and so forth) are the same or different across groups. Put simply, if we were to rank the 32 sounds according to their mean ratings of annoyance for misophonics then again for controls, would we find these rankings to be similar? Across all 17 features, there is a large and statistically significant correlation between the cross-group ranking of the sounds (see Figure 4). That is, although misophonics tend to rate sounds more negatively (in absolute terms) the relative difference between the sounds (i.e., ranking them from least to worst) is largely preserved between both groups.

### Heterogeneity within misophonics

**Figure 5:**
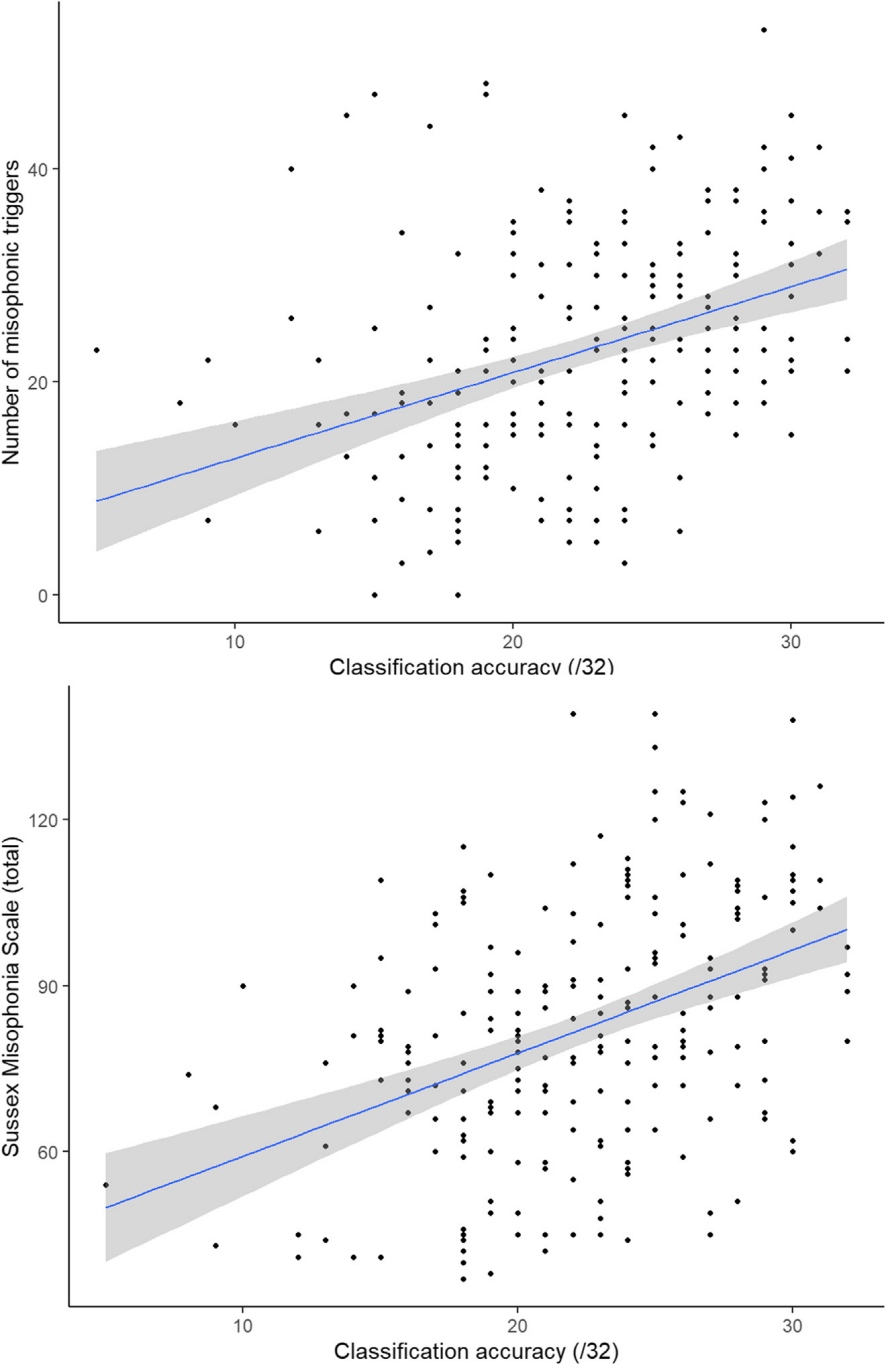
The ability to classify misophonics based on their response pattern to 32 different sounds (x axis) correlates with the number of misophonic triggers reported (top figure) and the severity of the misophonia (bottom) Each point is an individual misophonic participant.

This rich dataset offers opportunities for exploring individual differences among misophonics. As a starting point, one can identify misophonics who are easy or hard to classify according to the number of sounds for which they were correctly classified as having misophonia (a score from 0 to 32). For the misophonics, the mean number of sounds that led to an accurate group classification was 22.17 and S.D. was 5.11 (range = 5 to 32). These differences amongst misophonics in classification performance were significantly correlated with self-reported severity and breadth of misophonia from data collected in a previous session using the Sussex Misophonia Scale, SMS.^21^ The Pearson’s correlations between classification accuracy and the number of triggers or total questionnaire score were r = 0.381 and 0.417 respectively (p < 0.001), as shown in Figure 5. Figure S13 shows heatmaps for categorical differences in severity, comparing the transition from non-misophonia to moderate misophonia, and from moderate to severe misophonia (based on the three groups identified in our prior research^14^). These results show that our machine learning approach is tracking real-world severity and, moreover, that misophonia severity is linked to a less specific profile (i.e., extending to more sounds).

### Differential diagnostic profiles: Within misophonia

**Figure 6:**
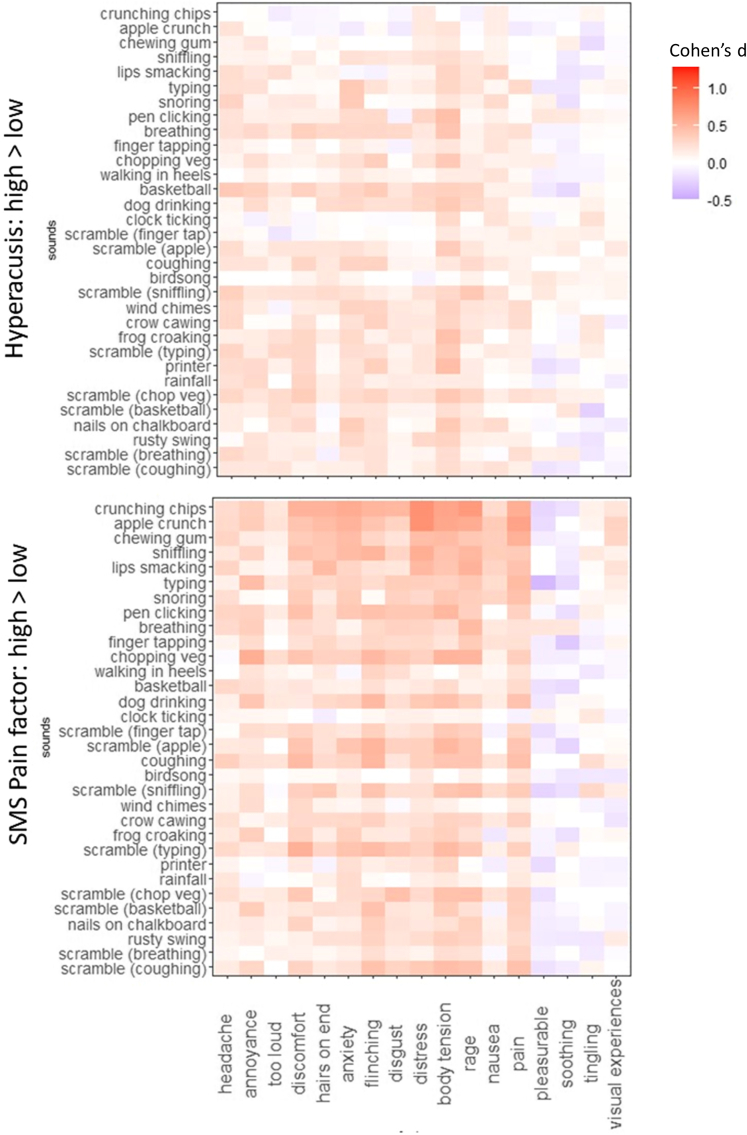
Heatmaps (showing Cohen’s d effect sizes) for 32 sounds (y axis) and 17 responses (x axis) for within misophonia comparisons, contrasting presence versus absence of hyperacusis (top) and high versus low scores on the SMS pain factor (bottom)

As our participants had been asked about the presence of other traits and co-morbidities, we can determine whether the pattern observed for misophonia is specific to misophonia. Effect sizes (Cohen’s d) were computed for different splits of the dataset. They are displayed as heatmaps for visualization purposes, and we trained a classifier (as before) to determine which sounds predict the various group memberships. The potential presence of hyperacusis within the misophonics was ascertained in two ways: either by directly asking them (based on^15^) or through a set of questions included within the SMS that ask about pain. These two different approaches yield different results, as shown in Figure 6, which depicts differences in responses as a function of self-reported hyperacusis (present vs absent) and pain (present vs absent). The results emphasize the importance of different responses (which are visualized as prominent columns), rather than different sounds. Those misophonics who agree to the hyperacusis question went on to give higher responses for “body tension” (across most sounds) whereas those who agreed to the “pain” questions on the SMS went on to give higher responses for headache and pain (across most sounds)—noting that these latter two features are not the strongest indicators of misophonia per se (see Figure 1 for example). The association between pain measures is not entirely trivial given that the measures were given months apart and differed substantially in method (trait-based questionnaire versus state-based responses to specific sounds). For classifiers trained to predict high/low pain for misophonics as reported in the SMS questionnaire, these could predict group membership above chance (p < 0.05) for 20 sounds with 12 remaining significant after FDR correction: sniffling (AUC = 0.672), pen clicking (AUC = 0.662), breathing (AUC = 0.658), rusty swing (AUC = 0.641), crunching chips (AUC = 0.639), chewing gum (AUC = 0.638), typing (AUC = 0.634), nails on chalkboard (AUC = 0.628), clock ticking (AUC = 0.625), basketball (AUC = 0.625), chopping vegetables (AUC = 0.620), and finger tapping (AUC = 0.618). For classifiers trained to predict self-reported hyperacusis, these could predict group membership above chance (p < 0.05) for 13 sounds with six remaining significant after FDR correction: scrambled typing (AUC = 1.000), finger tapping (AUC = 0.649), basketball (AUC = 0.629), scrambled coughing (AUC = 0.624), scrambled apple crunch (AUC = 0.622), and typing (AUC = 0.613). In summary, we show that co-morbid traits of hyperacusis and sound-induced pain have a different sound-response profile to misophonia and that the phenomenological cartography approach is sensitive to different forms of sound intolerance.

### Differential diagnostic profiles: Within non-misophonics

**Figure 7:**
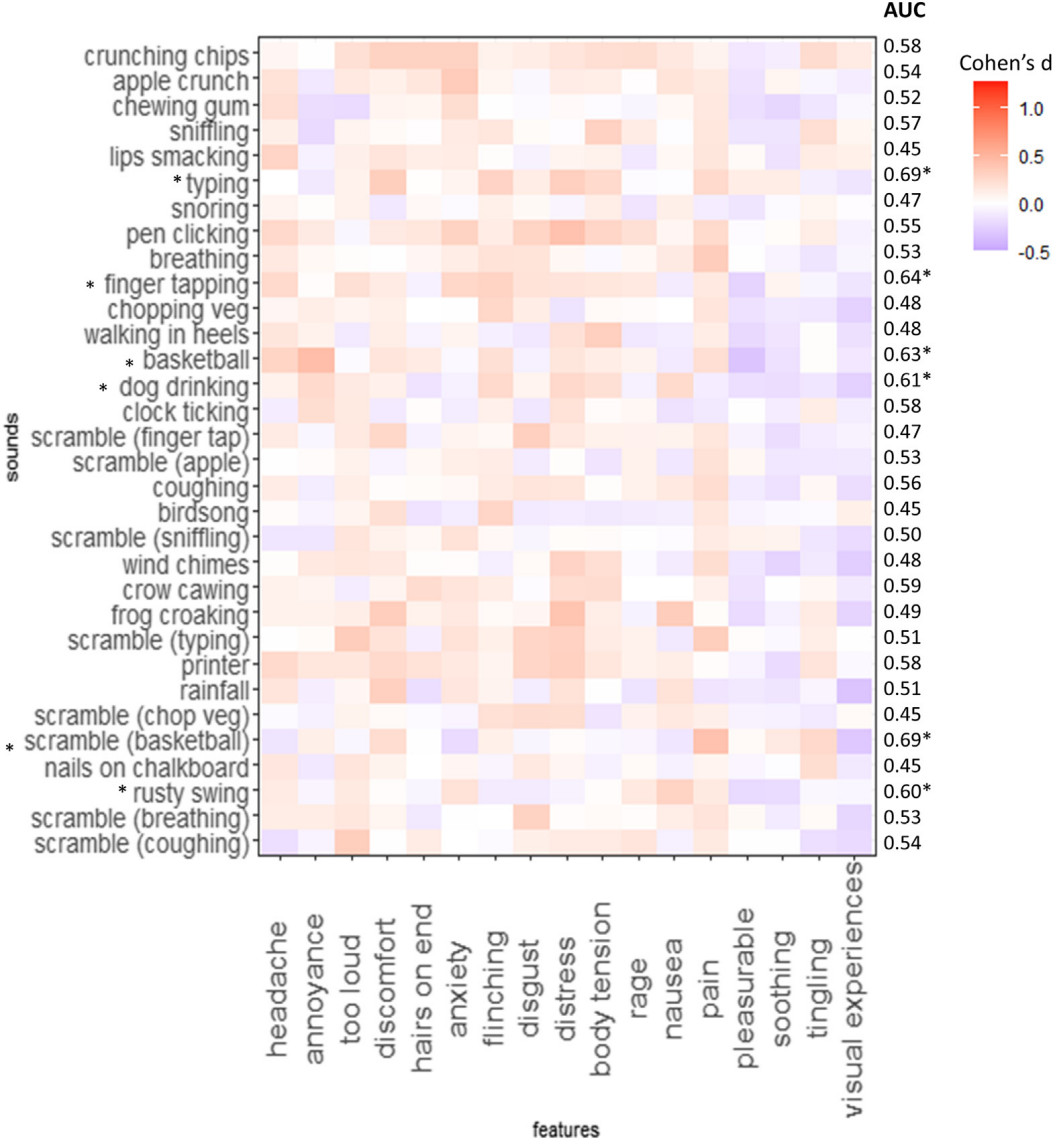
Heatmaps (showing Cohen’s d effect sizes) for 32 sounds (y axis) and 17 responses (x axis) for comparisons within non-misophonics, contrasting high versus low AQ scorers

With regard to individual differences amongst the non-misophonic controls, the effect sizes are generally less extreme. High versus low autistic traits (measured by the AQ) have some similarities with misophonia in that the four responses depicted on the right of the heatmap in Figure 7 showed the same reversed “blue” profile as misophonics (pleasurable, soothing, tingling, visual experiences). But, unlike misophonia, the top-to-bottom ordering of effect sizes was not apparent and—instead—there was a pervasive low-level negativity toward most sounds. The most discriminating sounds between high v. low AQ were in descending order: scrambled basketball (AUC = 0.692), typing (AUC = 0.690), finger tapping (AUC = 0.637), basketball (AUC = 0.631), dog drinking (AUC = 0.612), and rusty swing (AUC = 0.601). These were all significant with the top four surviving FDR correction.

Neither the data splits of high v. low sensory sensitivity (using the GSQ) or high v. low interoceptive awareness (using the MAIA) produced any significant results that survived FDR correction (see Figures S14 and S15).

### Comparison of misophonia and ASMR responders

A separate set of N = 254 participants were recruited and given questionnaires relating to misophonia (SMS)^21^ and ASMR (ASMR-15).^23^ In addition, they completed the phenomenological cartography study above with the original 32 sounds plus the addition of a further set of 8 ASMR triggers (e.g., whispering). There was no significant correlation between the ASMR-15 and the SMS scores (r = 0.113, p = 0.072). We applied the aggregated classifier from the previous sample (pooled from 32 sounds and 17 responses) to predict the misophonia status of these new participants. The probability of being classified as misophonic (from the machine learning algorithm) was correlated with the SMS scores (r = 0.399, p < 0.001) but not the ASMR-15 scores (r = −0.089, p = 0.159). The differences in correlation were significant (t(251) = 6.368, p < 0.001).

**Figure 8:**
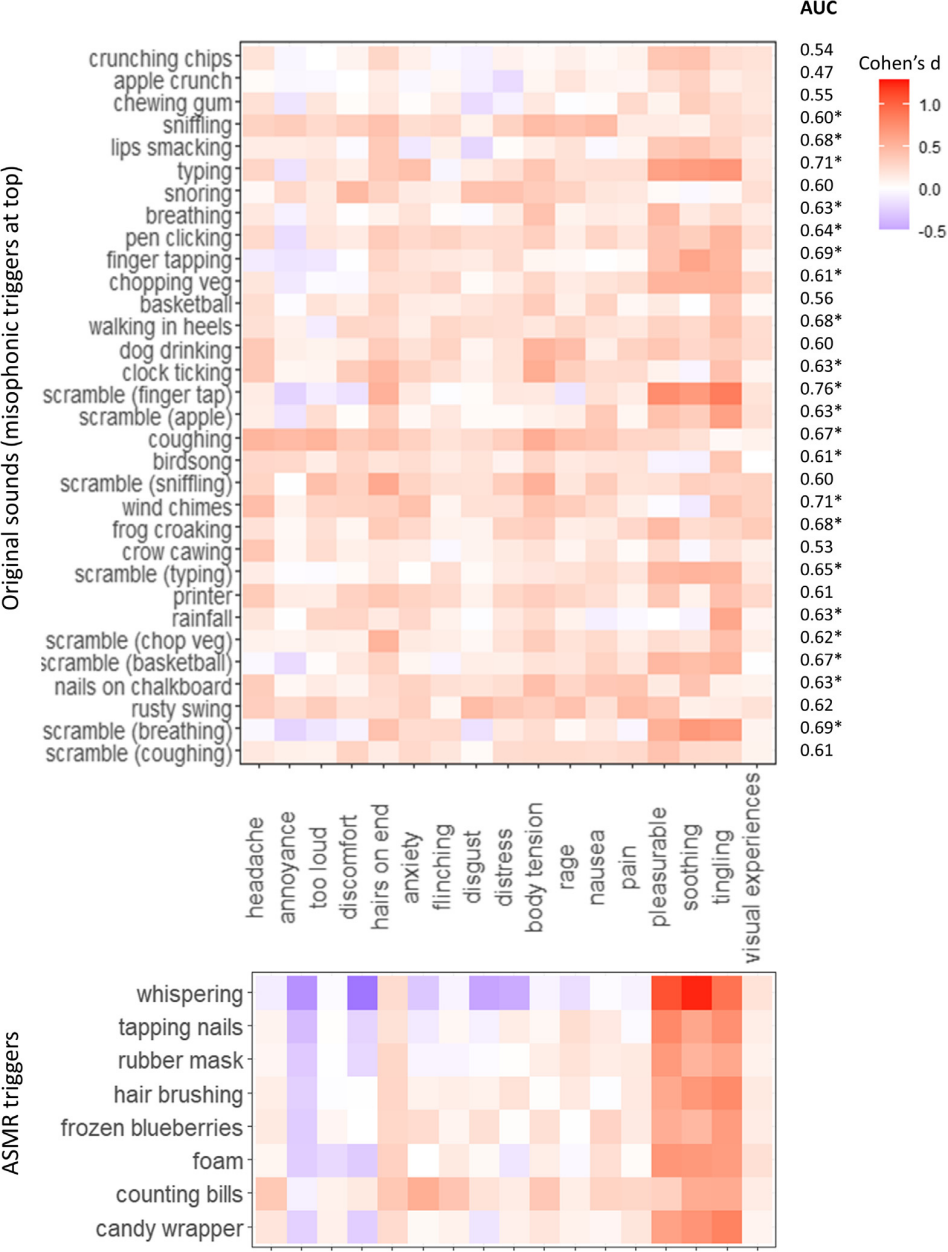
Heatmaps (showing Cohen’s d effect sizes) for (top) 32 sounds (y axis) and 17 responses (x axis) and (bottom) the eight ASMR triggers, contrasting ASMR responders and non-responders (excluding misophonics)

Excluding misophonics (i.e., those with SMS scores greater than the diagnostic threshold of 50.5), we created a separate phenomenological cartography heatmap contrasting high and low ASMR groups, by applying a mean ASMR-15 value of 2.5 (1–5 scale) to divide the groups. This gives a mean ASMR-15 score of 3.75 in the high group which is comparable with other studies of ASMR responders (3.49 in^24^ and 3.73 in^23^). The ASMR heatmap for the main set of 32 sounds is shown in Figure 8. The strongest group differences are found for the responses of soothing, tingling, and pleasurable, and were most strongly elicited by the scrambled sounds. ASMR status could be classified above chance from 21 sounds (after FDR correction) suggesting that ASMR, as with misophonia, is not limited to its traditionally conceptualized triggers. The eight ASMR triggers shows the same basic pattern (albeit with far larger effect sizes). These sounds also tended to be rated lower in annoyance and discomfort by ASMR responders relative to non-responders. Figure S16 shows the corresponding data for misophonics in this sample (with lower effect sizes and a pattern more similar to that documented previously for misophonia e.g., Figure 1), and those reporting both ASMR and misophonia (who tend to resemble the ASMR profile). In summary, we show that misophonia and ASMR have distinctive profiles with very little in common.

## Discussion

There are significant individual differences in the ability to tolerate (or enjoy) certain sounds which, in extreme situations, impacts on quality of life enough to require intervention. We developed an approach, which we term phenomenological cartography, to map out the sound-response profile of misophonia. We use this as a predictive tool by applying machine learning to classify participants. We demonstrate that, despite heterogeneity, there is a distinctive pattern linked to misophonia that extends across most sounds (but is clearly more extreme for certain sounds). There are individual differences within this profile: more severe misophonics are easier to classify, and the presence of sound-induced pain and/or hyperacusis within the misophonics can be considered a separate “layer” superimposed on the map (for further discussion of hyperacusis subtypes^25^). We further show that the misophonic sound-response pattern is different from the sound-response profile linked to the broad autism phenotype or ASMR. The former is linked to repetitive sounds (e.g., typing, basketball dribbling) and the latter to scrambled sounds, whispers, and so forth. In effect, phenomenological cartography enables us to dissect a complex set of subjective sound-response relationships into distinct cognitive profiles. This is important for driving scientific theory and for real-world practice (differential diagnosis and possibly treatment).

Misophonia is defined as a disorder of decreased tolerance to specific sounds,^1^ but our research challenges us to rethink the idea of specificity. Specificity could be taken to mean that a minority of sounds evoke misophonic responses against a backdrop of otherwise normal sound tolerances. Alternatively, specificity could be taken to mean that misophonic sounds are highly idiosyncratic or personal. Our data is incompatible with these interpretations but supports, instead, a weaker conclusion that some sounds are far worse for people with misophonia than others. Importantly, this sits against a backdrop of generalized sound intolerance and a high level of agreement (across individuals and groups) about which sounds are aversive. Similarly, there is a broad range of responses linked to misophonia although some (e.g., body tension, rage) were found to be more important than others (e.g., nausea, hairs on end). If specificity were the hallmark of misophonia then it is also puzzling to find an inverse relationship between specificity and severity: more severe misophonics have a less specific profile. In effect there are two key properties of misophonia that need to be explained: why there is a pervasive pattern of sound intolerances (generality) and why some sounds are worse than others (weak specificity). Whether a single explanation can account for both observations is unclear and is discussed further later in discussion.

It is conceivable, at least in theory, that specificity is an emergent property of generality such that there is a “gain” or amplification of the normal hierarchy of sound intolerances^26^—i.e., the degree of worsening in misophonia may be related to how aversive they are to begin with. We provide evidence that misophonics and controls do rank sounds similarly (across 17 rating scales) which speaks to this idea. However, there are important exceptions that suggest a more complex picture. To give two examples. “Rusty [creaking] swing” and “nails on chalkboard” are strongly disliked by both groups but the relative difference between the groups is marginal (as noted elsewhere^2^). On the other hand, crunching chips are far more misophonic than the sound of coughing (despite the latter having clear negative connotations). It is likely that the degree of aversion to sounds reflect a complex evaluation of multiple sources of information including its meaning (semantic content, social context, and so forth) and psychoacoustic properties. Changing the meaning of a sound (e.g., by presenting it with an incongruent video^27^) or making it harder to identify (e.g., presenting it against noise^28^) reduces the gap between misophonics and controls although it does not necessarily eliminate it (as shown also for our scrambled sounds). But whether a single dimension can predict the relative ranking of sounds (misophonic > control) remains to be shown.

Here we also tested whether a psychoacoustic feature linked to sound unpleasantness (frequencies between 2500 and 5500, Hz and 1–16 Hz temporal modulations^2^) predicts ratings from misophonics and non-misophonics. Our own dataset has the advantage of multiple rating scales but the disadvantage of a low number of sounds. But we also analyzed the dataset of Hansen et al.^6^ that is complementary to our own (many more sounds albeit rated in a more limited way). In neither dataset do we find evidence of a relationship between this psychoacoustic feature and ratings of natural sounds. However, we do find evidence of a relationship with our unrecognizable scrambled sounds (and so we speculate that many of the natural sounds in the original study by Kumar et al..,^2^ may also have been hard to recognize). As we only focus on this one psychoacoustic feature it remains a possibility that other (unknown) psychoacoustic properties are relevant to misophonia. It may also be the case that the psychoacoustic properties of sounds are more relevant for autism or ASMR than they are for misophonia. In ASMR it was the unrecognizable sounds that were most potent which suggest a reliance on sound properties over meaning. The repetitive sounds found to be predictive of autism extended to both natural and scrambled sounds, pointing to the same conclusion.

Misophonia has been found to be co-morbid with autism,^29^ hyperacusis,^14^^,^^30^ and ASMR.^11^^,^^12^^,^^13^ It is important to speculate how these co-morbidities can co-occur if they have different sound-response profiles. One possibility is that the profiles are layered on top of each other (i.e., additive in statistical terms). For example, someone with misophonia and autism may have both particularly strong responses for misophonic triggers (chewing, and so forth) and those linked to autism (tapping, and so forth) against a backdrop of general sound intolerance that acts as a common denominator to both conditions. Hyperacusis could be understood in a similar way, albeit manifesting itself more in terms of a characteristic response profile (e.g., pain) than sound specificity. That is, it is conceivable that sound intolerances in misophonia, hyperacusis, and autism all stem from a general sensory hyper-sensitivity that, whilst strongest for sounds, is by no means limited to it.^31^^,^^32^^,^^33^ In effect, generalized sensory hyper-sensitivity may be a common seed from which somewhat different profiles emerge during development. A comorbidity between misophonia and ASMR is harder to understand as layering of different profiles because they would tend to cancel each other out (e.g., ASMR responders report high pleasure and soothing, and misophonics report the opposite). Indeed, our misophonia classifier does not predict ASMR-15 scores (the correlation is, if anything, negative). The self-report questionnaires used in our study hint at a weak positive relationship. This may reflect superficial similarity (sound over-responsiveness) not borne out by detailed assessment. ASMR and misophonia may also arguably differ in terms of whether the response is primarily somatosensory or affective/interoceptive respectively.^10^

One surprising difference between our groups was that misophonics reported less “visual experiences.” Further research is needed to understand how participants interpreted this term, but we speculate that it is related to mental imagery (e.g., because visual experience ratings here were uniformly low for scrambled sounds which can’t be easily imagined). By contrast, such a pattern would not be expected for sound-vision synaesthesia, where visual experiences are elicited by all sounds, and where these experiences are very rare.^34^ An fMRI study also found that misophonics have less activity (compared to non-misophonics) in the visual ventral stream when listening to misophonic triggers,^5^ which is consistent with our results. Further research is needed to understand whether this is a general difference in the capacity to generate visual images, or if it is context specific. It may be that when listening to sounds, misophonics divert attention inwards (to the reaction in their body) whereas other people imagine the source of the sound. If so, this could potentially be developed as a target of treatment for misophonia, in the form of strategic reorienting.

In sum, the novel approach of phenomenological cartography has revealed important insights into the likely mechanisms of misophonia (it is linked to broad sound intolerance), the differential diagnoses of other atypical sound sensitivities (e.g., requiring minimal translation to be used cross-culturally), and possible treatments for misophonia (visualizing the source).

### Limitations of the study

Machine learning is commonly criticized for the lack of interpretability of results (a “black box” approach) and the risk of over-fitting (inflated performance due to, e.g., detecting confounds). Both are valid limitations of the present study, although we made attempts to mitigate them. Interpretability was aided by performing more conventional analyses in parallel (e.g., heatmaps derived from mass univariate analyses) and separate train and test resamples guards against over-fitting. Other choices of machine learning algorithms were not compared. Finally, whilst we took advantage of having rich phenotypic information about our participants (enabling us to split the dataset in different ways) further research is needed to validate differential diagnoses on clinically defined autism and hyperacusis. The generalizability to other demographics also needs to be established, including cross-culturally.

## STAR★Methods

### Key resources table

**Table:** 

REAGENT or RESOURCE	SOURCE	IDENTIFIER
**Deposited data**		

Raw and analyzed data	This paper	https://osf.io/27fgv/

**Software and algorithms**		

Time-domain scrambling of audio signals in Matlab	Ellis^35^	https://www.ee.columbia.edu/∼dpwe/resources/matlab/scramble/#1
NSL (Neural Systems Laboratory) auditory-cortical Matlab toolbox	Chi, Ru and Shamma^36^	http://nsl.isr.umd.edu/downloads.html
VSURF, Variable Selection using Random Forests,^22^	Genuer et al.^22^	https://journal.r-project.org/archive/2015/RJ-2015-018/index.html

**Other**		

Pre-registered methods and analysis plan	This paper	https://osf.io/27fgv/
Sound stimuli	This paper	https://osf.io/27fgv/

### Resource availability

#### Lead contact

Further information and requests for resources and materials may be directed to and will be fulfilled by the lead contact, Prof. Jamie Ward (jamiew@sussex.ac.uk).

#### Materials availability

• Sounds used and created in this research are available for download and re-use (without permission) from https://osf.io/27fgv/.

### Experimental model and subject details

This study was approved by the Cross-Schools Science and Technology Research Governance and Ethics Committee of the University of Sussex, UK.

#### Sample comparing misophonia and non-misophonic controls

A total of 418 participants completed the task and were divided into two groups: a non-misophonic control group (N = 196; mean age = 33.08, S.D. = 14.29; gender = 140 female, 55 male, 1 non-binary) and a misophonic group (N = 222; mean age = 41.56, S.D. = 14.32; gender = 175 female, 42 male, 5 non-binary). Misophonia is equally prevalent across sexes^37^ and the over-representation of women in our samples is attributed to a recruitment bias. This sample size is sufficient to detect Cohen’s d of 0.3 and above (with power = 0.85) in univariate analyses. Details of their recruitment and assignment into groups are documented elsewhere,^14^ but briefly restated here. The Sussex Misophonia Scale,^21^ rather than recruitment source, was used to determine group status. Here we used a clustering method based on the five factors of this scale to identify three groups: non-misophonic, moderate misophonia, and severe misophonia. The latter two groups are collapsed in the present study when performing a binary classification, and we note that the binary group division closely corresponds to the published cut-off value of >50.5 using this measure.^21^ The mean total SMS score of non-misophonics was 9.026 (S.D. = 9.006) and for misophonics it was 82.112 (S.D. = 22.767).

#### Sample comparing misophonia and ASMR responders

Participants were recruited from the psychology undergraduate population at the University of Sussex, but with some additional participants recruited from our misophonia database and an ASMR discussion forum (on reddit) to ensure we received sufficient high responses on these measures. Misophonia and ASMR tendencies were determined by the relevant measures rather than self-declaration or recruitment source. A total of N = 254 participants completed all relevant parts of the study (sound ratings, ASMR-15, SMS) and took a sufficiently long time to complete the sound task (>10 min). The mean age was 23.224 years (S.D. = 8.165) with 202 women, 45 men, and 7 non-binary.

### Method details

#### Sound stimuli

The final set of sound stimuli consisted of a core set of 32 sounds, together with an additional set of 8 sounds (ASMR triggers) which were given to a second sample.

The core set of 32 sounds selected for this task were downloaded from a free sound database (https://freesound.org/) and spliced or concatenated to be 15 s long. Their selection was motivated by two previous studies investigating the reactions of misophonics and controls to natural sounds from different categories, and not limited to known misophonic triggers.^6^^,^^38^ Our sound clips were eight everyday sounds from each of three categories; these are human-made sounds originating from the nose or mouth (i.e., chewing apple, eating crisps, chewing gum, lip smacking, coughing, snoring, breathing, sniffling), other human-made sounds (i.e., playing basketball, chopping vegetables, clicking a pen, finger tapping, nails on chalkboard, swinging on swing set, typing, walking in heels), non-human sounds (i.e., birds singing, clock ticking, crow cawing, dog drinking water, frog croaking, printer, rainfall, wind chimes).

We also present a new category that are used as control sounds; these sounds were created by using four of the human oral/nasal and four of the other human sounds (i.e., apple eating, breathing, coughing, sniffling, basketball, chopping vegetables, finger tapping, typing), and scrambling them in time. Scrambling was done by selecting 15 ms snippets from the sound and randomly re-arranging these within 400 ms from the original snippet location in the sound file using existing MATLAB (Mathworks Inc., Natick, MA) scripts.^35^ This method of scrambling preserves the original spectral content of a sound whilst removing local temporal structure, thus making it less recognisable whilst retaining its spectral similarity to the original sound.

The final selection of the 32 sounds was validated in two earlier pilot studies (N = 28 and N = 22 recruited from students at the University of Sussex), with natural sounds and scrambled sounds presented to different groups (i.e., so they did not prime each other). The pilot included several additional sounds that were subsequently discarded. The primary aim was to check that participants could recognise the natural sounds, and that the scrambled control sounds were not recognisable. Loudness and discomfort ratings were also collected for each sound using a 5-point Likert scale (none, a little, some, lots, extreme). Real sounds were on average highly recognisable (Mean recognisability = 87.4%, SD = 15.7%), whereas the scrambled sounds were on the whole not-recognisable (7.4% recognisability, SD = 12.4%). The loudness of 7 real sounds were adjusted by scaling intensity in Praat^39^ based on the average subjective loudness rating from the pilot data, and the corresponding scrambled sounds were re-scrambled from the loudness-adjusted originals.

The eight ASMR triggers were selected from an initial pool of 16 after piloting them on a separate group (N = 24; mean age = 26.5 years, SD = 5.65; 15 female, 8 male, 1 non-binary). The pilot sample were recruited from an ASMR reddit forum, all self-identified as having ASMR, and the average score on the ASMR-15 was 3.86 (S.D. = 0.43). The sound clips for the pilot lasted 1 min. Participants were asked after each sound whether they experienced ASMR. They were also asked to press the spacebar when they first began to experience ASMR (start time) and ‘enter’ when their ASMR peaked (peak time). The eight most reliable ASMR triggers, eliciting a response in at least half of the pilot sample, were selected. In the selected stimuli, the average start time for onset of ASMR was 21.79s (S.D. = 5.09, range = 17–30) and the average peak time was 35.30 (S.D. = 6.06, range = 26–46). The original sound files were trimmed to 30s each for the main study (i.e., after ASMR onset and close to peak). A full list of triggers, and the pilot data, are included in Table S1.

#### Procedure

Participants were required to use a computer screen, with headphones or a computer speaker. They were initially played a musical clip and were asked to adjust the loudness to be as loud as possible without causing any discomfort. They were instructed to maintain this loudness for the duration of the task. Participants listened to 32 sound clips (or 40 in the follow-up study) once or repeatedly if necessary. These were played in a random order. Participants then rated their reactions to each of these sounds. There were 17 descriptors used for the ratings and these were rated on a visual analog scale (ranging from 0 to 100) with endpoints marked as ‘none’ and ‘extreme’ (Figure S1). The descriptors were as follows: pain, rage, disgust, hairs-on-end, headache, flinching, tingling, soothing, pleasurable, annoyance, nausea, visual experiences, distress, body tension, anxiety, discomfort, too loud.

After completing the rating task, the participants took part in a set of other questionnaires. For our main study (N = 418), these measures are written-up and analysed separately^14^ although we make additional use of some of them here: namely, the autism spectrum quotient AQ,^40^ Glasgow Sensory Questionnaire GSQ,^18^ and the Multidimensional Assessment of Interoceptive Awareness MAIA,.^19^ For our second sample (N = 254), the main measures of interest are the previously described SMS and ASMR-15.^23^ The ASMR-15 consists of fifteen questions requiring an answer on a five-point Likert scale (Completely untrue for me; Somewhat untrue for me; Neither true nor untrue for me; Somewhat true for me; Completely true for me). They were given the generic instructions: “This survey is looking at how certain stimuli affect you. Some individuals experience intense physical and emotional responses upon hearing particular sounds. These sensations and feelings can be pleasant or unpleasant. Sounds such as whispering, crackling, tapping, or scratching may produce particular experiences described below. Using the scale, please indicate your level of agreement with each statement, upon hearing any of these, or similar sounds:” and then asked to consider it in the context of “When I hear certain sounds, such as whispering, crinkling, tapping…”. Example items include “It feels like goosebumps on the back of my head” and “The experience is blissful.” Items are averaged giving a 1–5 continuous scale.

### Quantification and statistical analysis

#### Machine learning

An aim of this analysis is to extract, in a data-driven way, a misophonic profile from the 32 sounds (creaking, crunching, etc.) x 17 responses (Rage, Disgust, Soothing, etc.). Initially each sound was analyzed separately to consider the possibility that different sounds have a different misophonic profile. The outcome of this stage is a ranked order of the 32 sounds according to their ability to classify misophonics from controls. The dataset consists of, for each sound, a set of 17 features (ratings for each of the dimensions) from all respondents together with category labels (misophonic, non-misophonic). A Random Forest classifier was trained using 10-fold cross validation and one tuned hyper-parameter (the number of features in each tree varying between 2 and 8). Random Forests have several advantages: they can be used where the number of features exceeds the number of examples without requiring data reduction, they have few hyper-parameters to tune, and the use of multiple decision trees protect against over-fitting. All features were rescaled using the max-min approach. The main dependent variable was accuracy (measured as area-under-curve, AUC) where 0.5 is chance and 1.0 is perfect classification. Specificity (proportion of misophonics correctly classed) and sensitivity (proportion of non-misophonics correctly classed) are noted. Permuted datasets (N = 1000) in which group labels are randomly shuffled were created to assess whether the AUC is significantly greater than chance (p < 0.05). When appropriate, FDR (False Discovery Rate), correction for multiple comparisons is also performed.^41^ The most important features that drive classification was determined using the R package, VSURF (Variable Selection using Random Forests)^22^ which has been shown to perform favourably compared to other solutions.^42^ This ranks the N = 17 features by their degree of importance for classification and we report, for interpretation purposes, those features that are not eliminated in the first step.

To analyze the degree to which the ability to classify a person as misophonic is sound-specific, one can apply cross-classification. For example, one can determine whether the ‘apple crunching’ classifier also be used to identify misophonic people’s responses to the ‘pen clicking’ sound. Here we took the trained classifiers from above and tested them on the response profile from all other sounds taking AUC as a measure of classification accuracy.

The extent to which the putative ‘misophonia profile’ is specific to misophonia or related to other conditions/traits is explored by splicing our participant groups in other ways. This is feasible because our participants completed a variety of other measures. Specifically, within our control (non-misophonic) group we split the group into high/low according to their scores on the AQ, GSQ and MAIA. For the AQ, we used the published cut-off of ≥ 23 for the ‘Broad Autism Phenotype’ and above^17^ (yielding groups of N = 57 and 139) and for the other measures a median split was taken in the absence of agreed cut-offs. Within the misophonics, we were interested in the co-morbid presence of hyperacusis. We had previously included a self-report question about hyperacusis (“Hyperacusis: When everyday sounds feel overwhelming, loud, intense, or painful that do not bother other people in the same way”), taken from a scoping review.^15^ There were N = 122 misophonics agreeing and N = 99 disagreeing that this statement applied to them. Another potential measure of hyperacusis, is the Pain factor of the SMS^21^ which consists of 4 questions answered on a 5-point Likert scale (never, hardly ever, sometimes, often, always). Here we divided participants according to those answering, on average, above or below the scale midpoint such that the high group was above ‘sometimes’ and the low group was at or below that point (82 and 139 participants respectively). For each of these splits of the dataset, the machine learning analysis procedure described above was re-run although we adopted 5-fold cross-validation to take into account the smaller sample sizes.

#### Psychoacoustic properties of the sounds

For several analyses, it was important to know more about the psychoacoustic features of our sound stimuli. We applied the mathematical model of Chi, Ru and Shamma^36^ on the spectral and temporal features of our sounds to generate a model of the cortical representation of sound filtered/tuned to different spectro-temporal modulations. This was done using the NSL (Neural Systems Laboratory) auditory-cortical Matlab toolbox (http://nsl.isr.umd.edu/downloads.html). Specifically, the cortical representation of each sound was represented as four dimensions: sound frequency (128 bins with centre frequencies ranging from 182 to 7143 Hz); spectral modulations (7 bins: 0.125. 0.25, 0.5, 1, 2, 4, 8 cycles per octave), temporal modulations (12 bins: +/- 32, 16, 8, 4, 2 and 1 Hz), and time (5 ms bins). Following the NSL code, the centre frequencies were calculated as follows: 440 ∗ 2 ˆ ([(0:128)-31]/24 (this covers around 5.3 octaves with 24 bins per octave, with 440 Hz being a central reference frequency for music). Following Kumar et al.^2^ we calculated, for each sound, the mean intensity between 2500–5500 Hz and 1–16 Hz temporal modulations (averaging over time and spectral modulations). These values were correlated against the mean group ratings for the same sounds. We also conducted the same psychoacoustic analysis of the N=125 sounds given to misophonics and controls by Hansen et al.^6^ and rated on the single dimension of discomfort. These analyses were not pre-registered and are a post-hoc exploration of the data.

Finally, we also used the derived cortical representations to better understand the effect that our scrambling procedure had on the sounds by comparing the original 24 sounds against their scrambled counterparts. A summary of this analysis is included in the supplemental information (Figures S2–S5).

## Data Availability

• All raw data has been deposited at Open Science Framework (https://osf.io/27fgv/) and is publicly available as of the date of publication. • All original code has been deposited at Open Science Framework (https://osf.io/27fgv/) and is publicly available as of the date of publication. • Any additional information required to reanalyze the data reported in this paper is available from the lead contact upon request.
