# Supplementary material for: A phenomenological cartography of misophonia and other forms of sound intolerance
Source: iScience. 2023 Feb 28;26(4):106299. doi: 10.1016/j.isci.2023.106299 (PMC10156615; doi:10.1016/j.isci.2023.106299)
Supplement: Document S1. Figures S1–S16 and Table S1 [file mmc1.pdf]

iScience, Volume *26*

Supplemental information

A phenomenological cartography of misophonia and other forms of sound intolerance

Nora Andermane, Mathilde Bauer, Ediz Sohoglu, Julia Simner, and Jamie Ward

Supplemental Information

Figure S1: Screenshot of the procedure for collecting multivariate responses to a single sound. These ratings are entered into a machine learning algorithm designed to classify participants as having misophonia or not. Related to STAR Methods (Procedure).

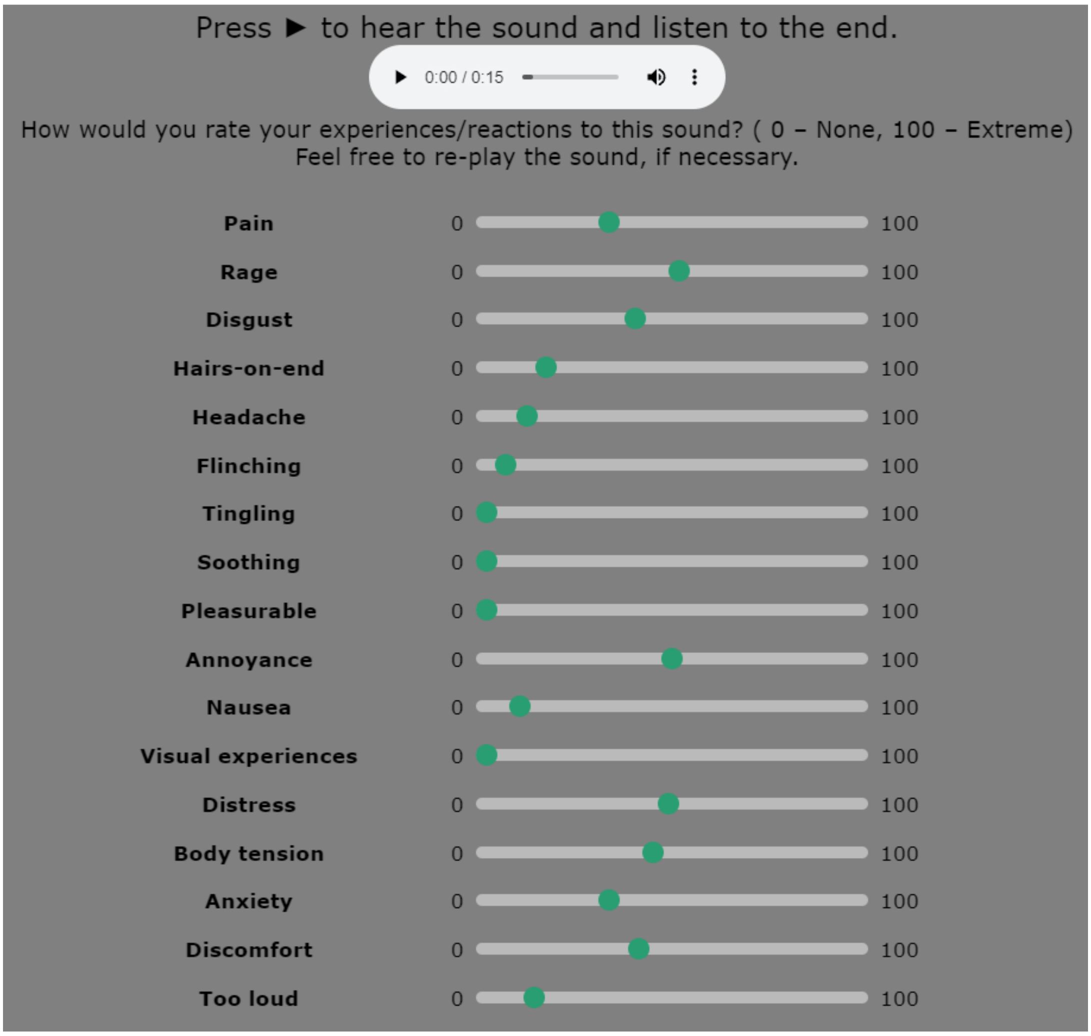

Figure S2: An example spectrogram (time on x-axis, frequency on y-axis) shown for one stimulus - finger tapping - before and after scrambling. Related to STAR Methods (Psychoacoustic Properties of the Sounds).

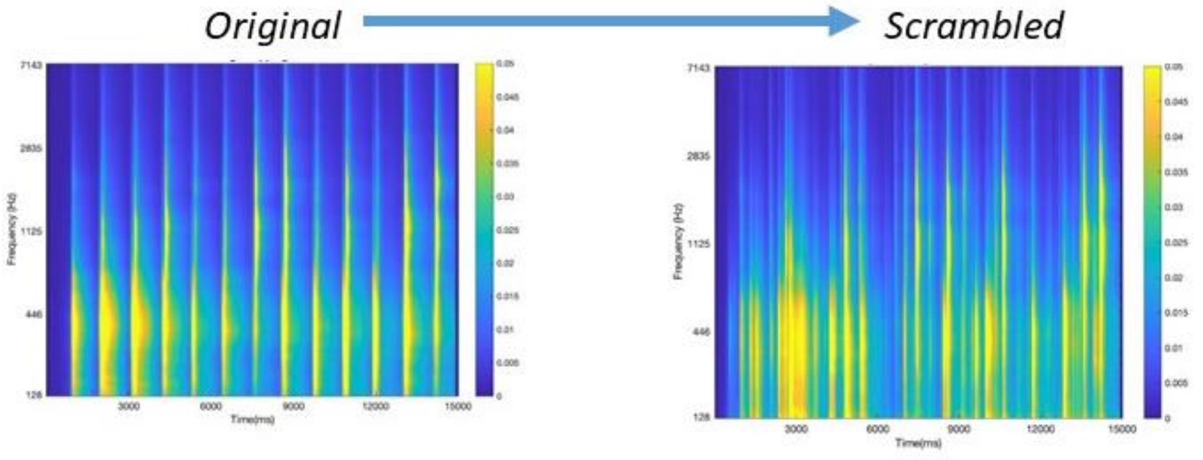

Figure S3. A comparison of the 24 environmental sounds used in the study together with the 24 equivalent sounds after scrambling. Scrambling reduces low frequency temporal modulations (+/- 2 Hz) but increases higher frequency temporal modulations (* p < .05). A 2x12 repeated measures ANOVA revealed a main effect of scrambling (F(1,23)=54.652, p < .001, eta2 = 0.704) and a significant interaction (F(1,253)=27.463, p < .001, eta2 = 0.544). Related to STAR Methods (Psychoacoustic Properties of the Sounds).

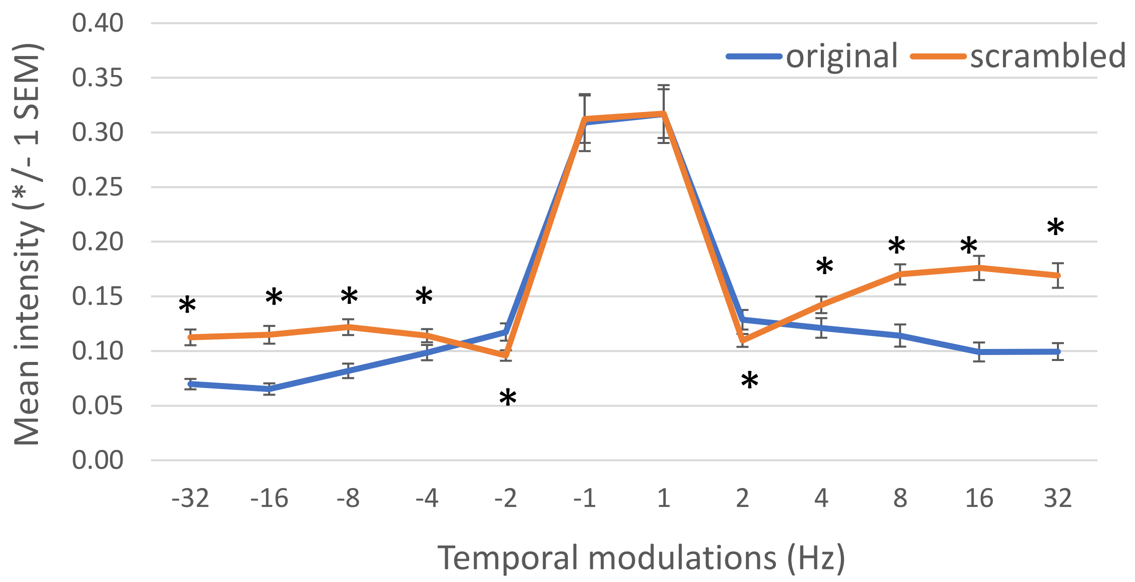

Figure S4. Scrambling has no significant effects on spectral modulations (comparing the 24 environmental sounds against their 24 scrambled counterparts). A 2x7 repeated measures ANOVA revealed no main effect of scrambling ($F(1,23)=0.029$, $p = .866$, eta2 = 0.001) and no significant interaction ($F(1,138)=1.833$, $p = .097$, eta2 = 0.074). Related to STAR Methods (Psychoacoustic Properties of the Sounds).

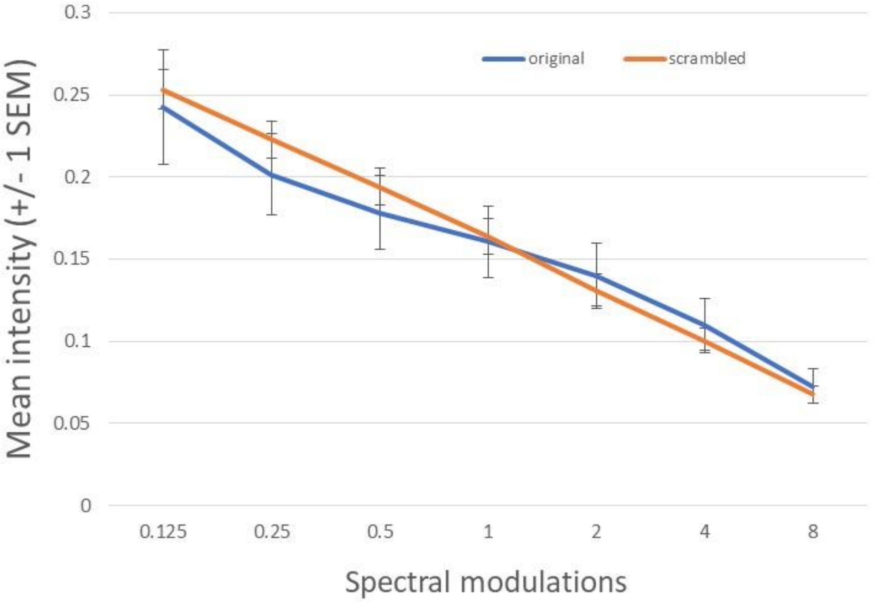

Figure S5. Scrambling has statistically small effects on the frequency profile (comparing the 24 environmental sounds against their 24 scrambled counterparts), although it is to be noted that scrambling systematically reduces the intensity across most frequency ranges. Related to STAR Methods (Psychoacoustic Properties of the Sounds).

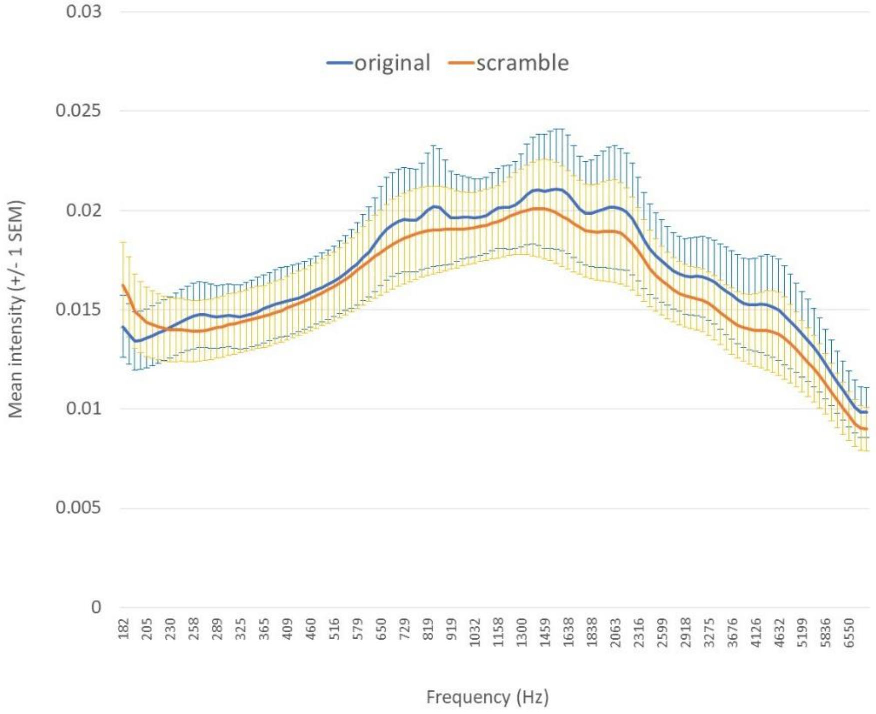

Figure S6. Dendrogram constructed from hierarchical clustering of the correlations between the 17 descriptors. Descriptors that branch low down are more similar (i.e. highly correlated) and those that branch higher up are more dissimilar. Two main branches are observed consisting of negative descriptors (left) and positive/neutral descriptors (right). Headache stands out as being more dissimilar to other negative descriptors. Related to Figure 1.

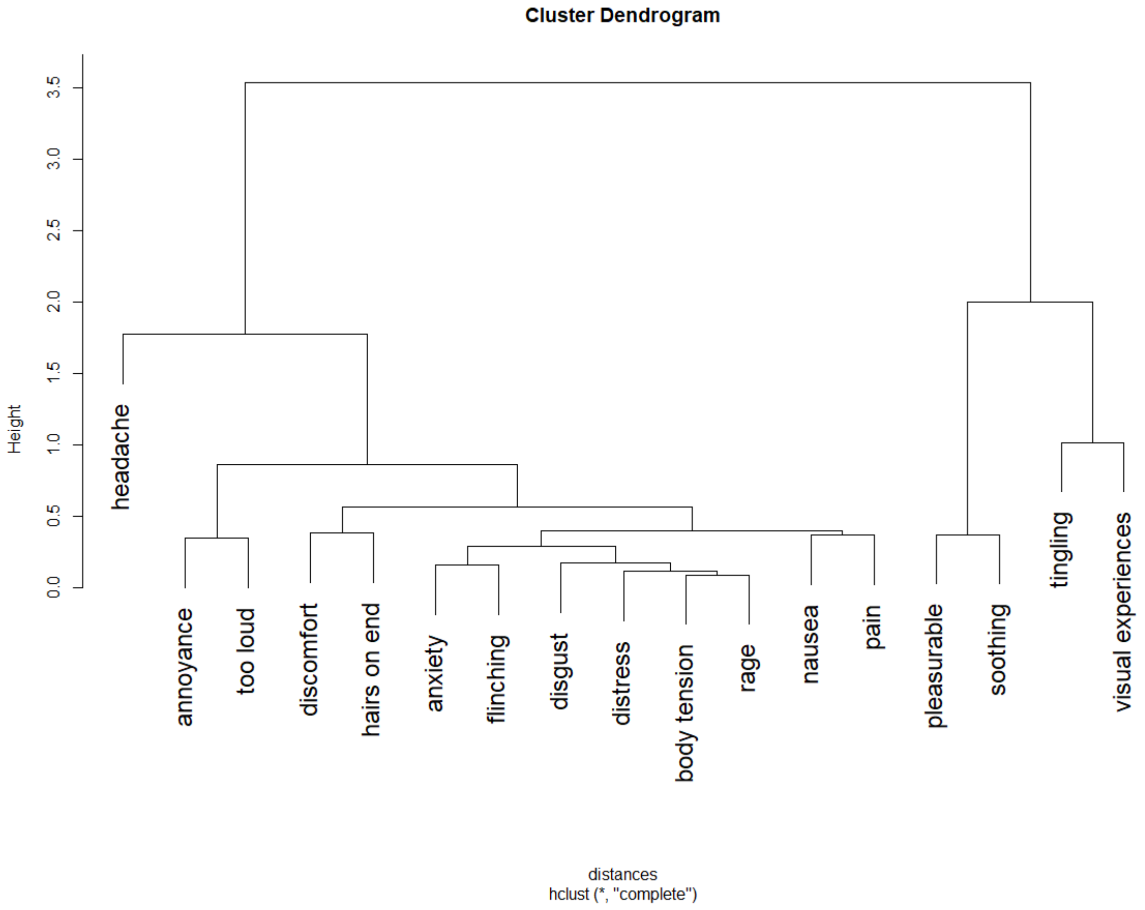

Figure S7. A reproduction of the same heatmap shown in Figure 1 (main paper) but here thresholded to only show cells where a significant group difference (p < .05 uncorrected) was observed. Note that the machine learning classifier takes into account all data, whether significant or not, so this figure is primarily for visualization and interpretation purposes. Related to Figure 1.

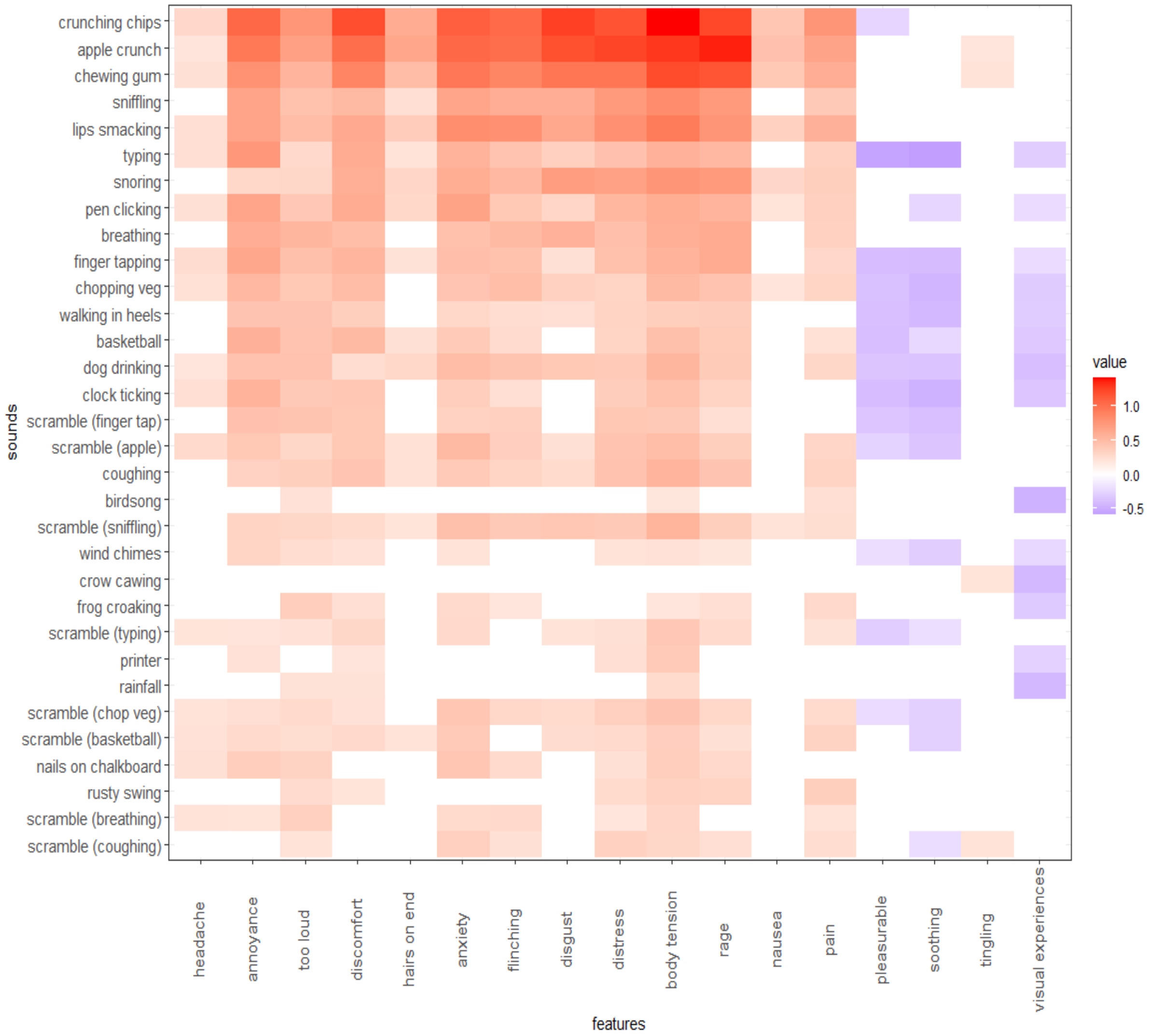

Figure S8. Mean ratings for the two groups (ranked by the mean for the misophonic group) for annoyance (top left), anxiety (top right), body tension (bottom left), and discomfort (bottom right). Error bars show +/- 1 SEM. Related to Figure 4.

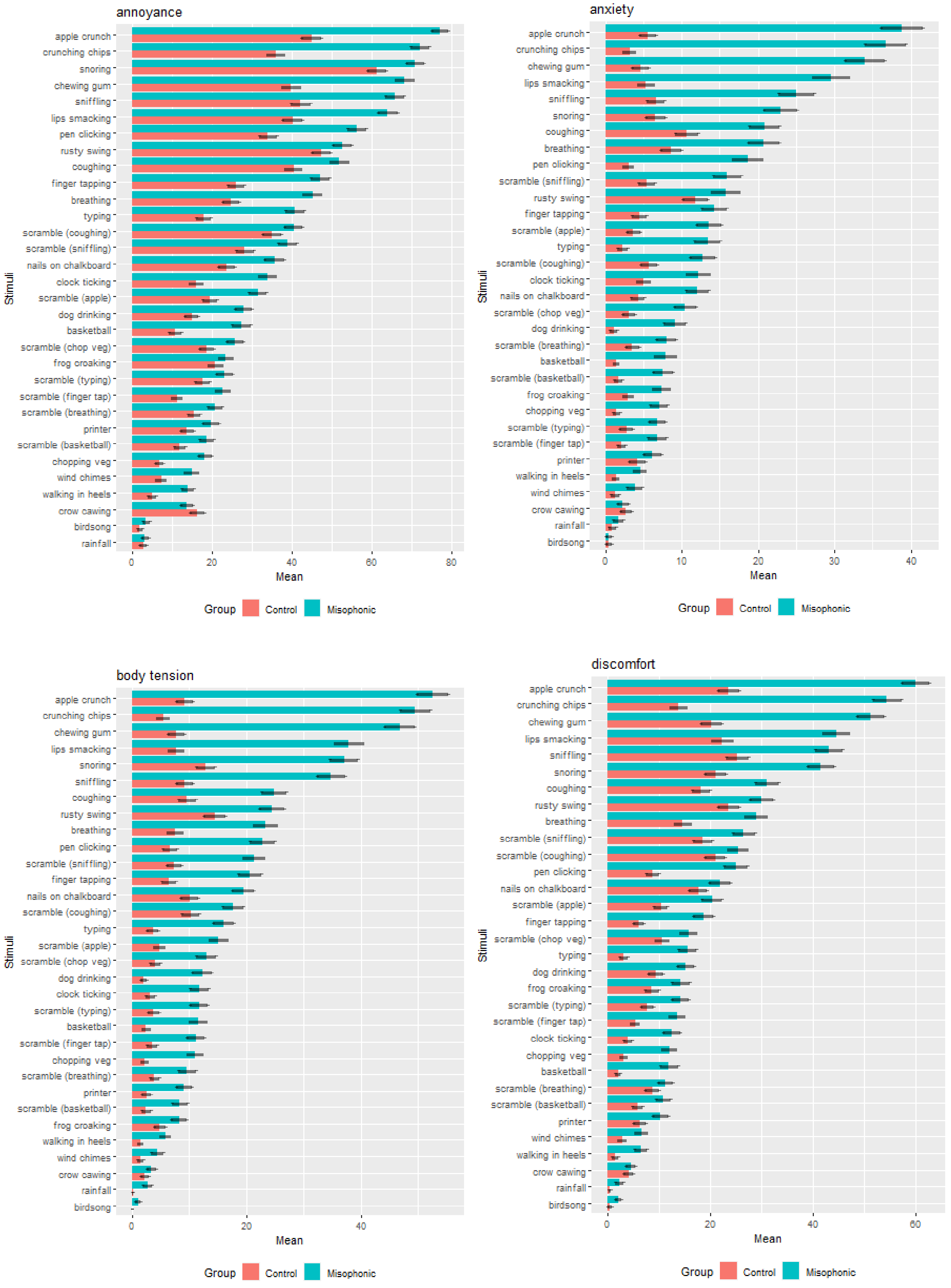

Figure S9. Mean ratings for the two groups (ranked by the mean for the misophonic group) for disgust (top left), distress (top right), flinching (bottom left), and hairs-on-end (bottom right). Error bars show +/- 1 SEM. Related to Figure 4.

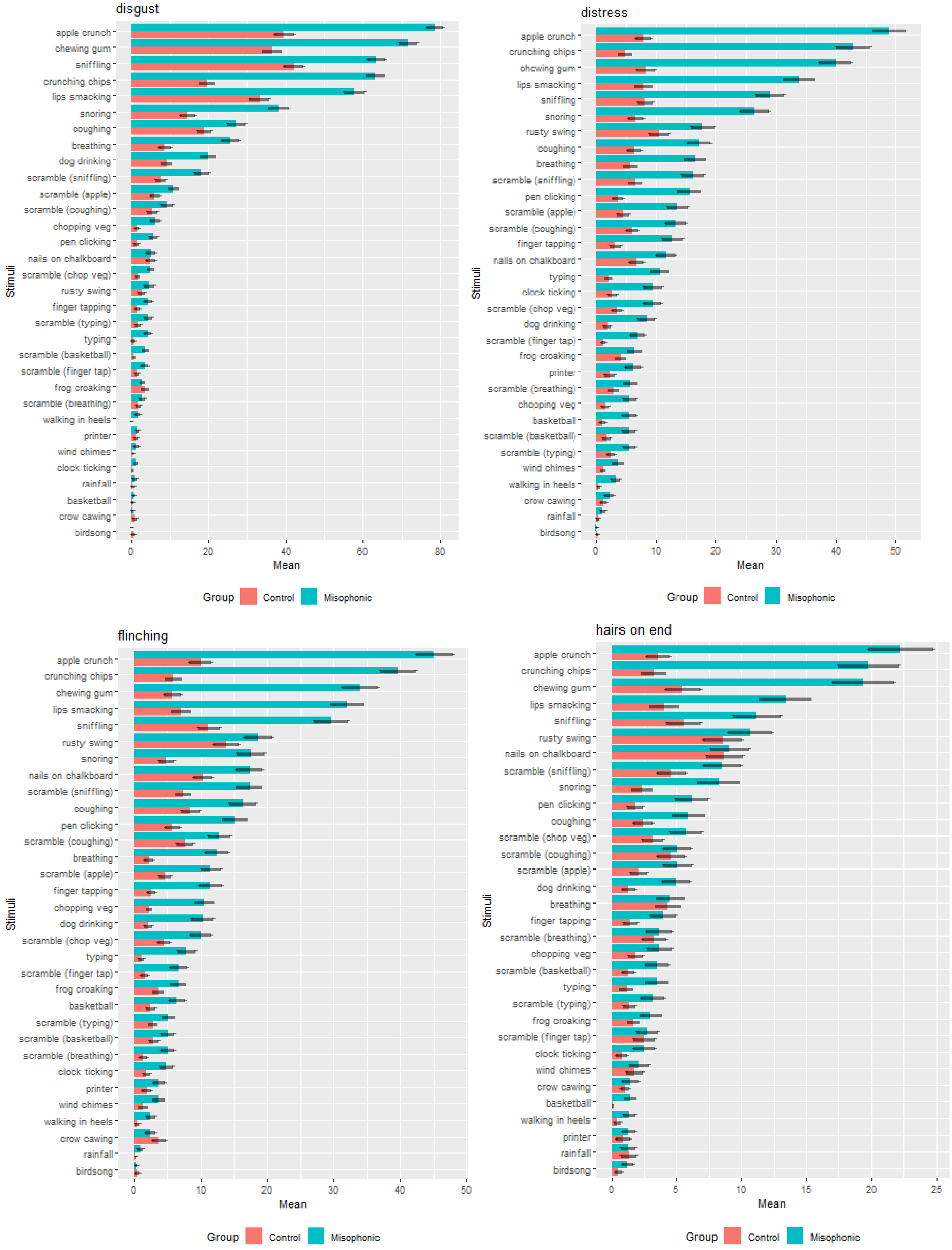

Figure S10. Mean ratings for the two groups (ranked by the mean for the misophonic group) for headache (top left), nausea (top right), pain (bottom left), and pleasurable (bottom right). Error bars show +/- 1 SEM. Related to Figure 4.

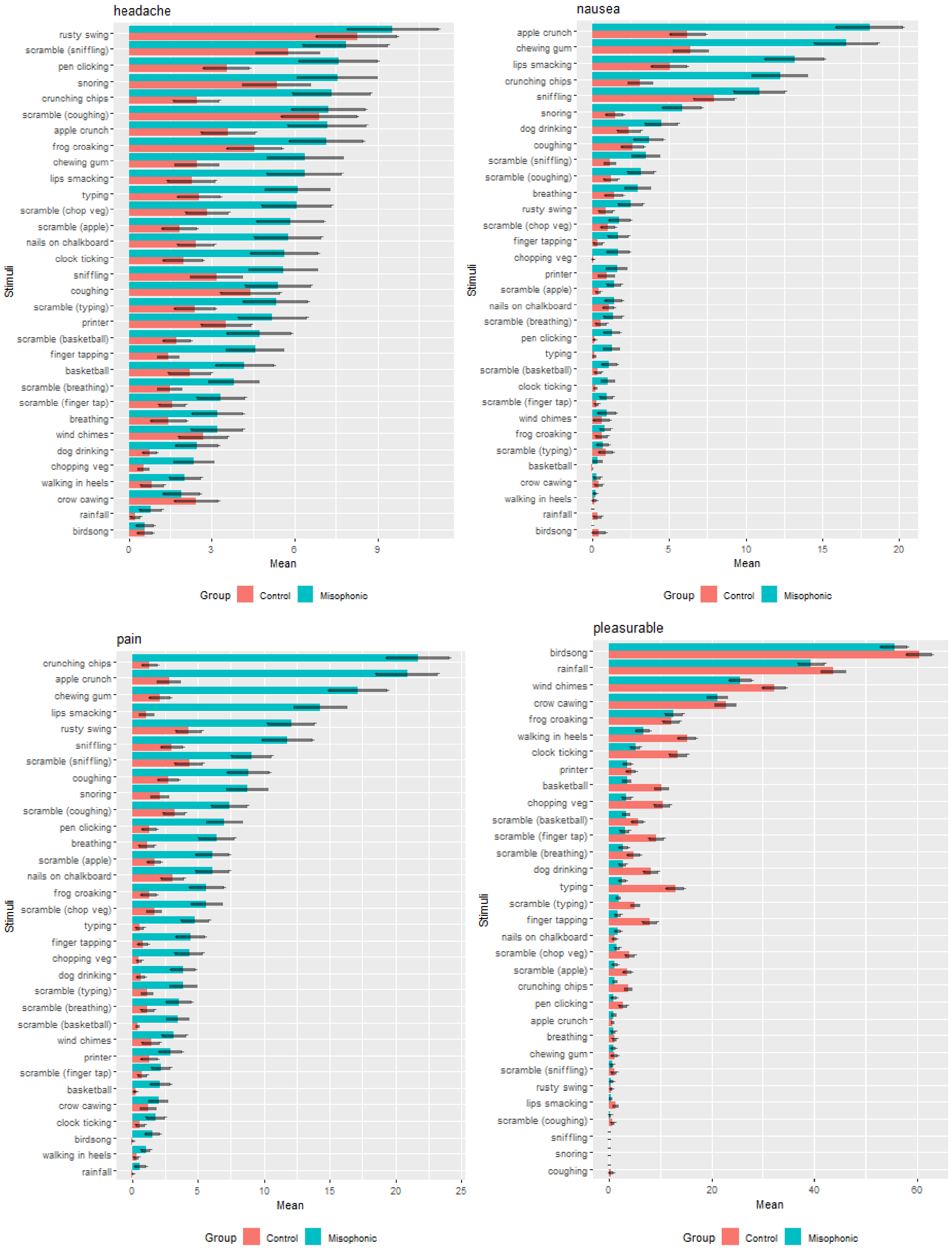

Figure S11. Mean ratings for the two groups (ranked by the mean for the misophonic group) for rage (top left), soothing (top right), tingling (bottom left), and too loud (bottom right). Error bars: +/- 1 SEM. Related to Figure 4.

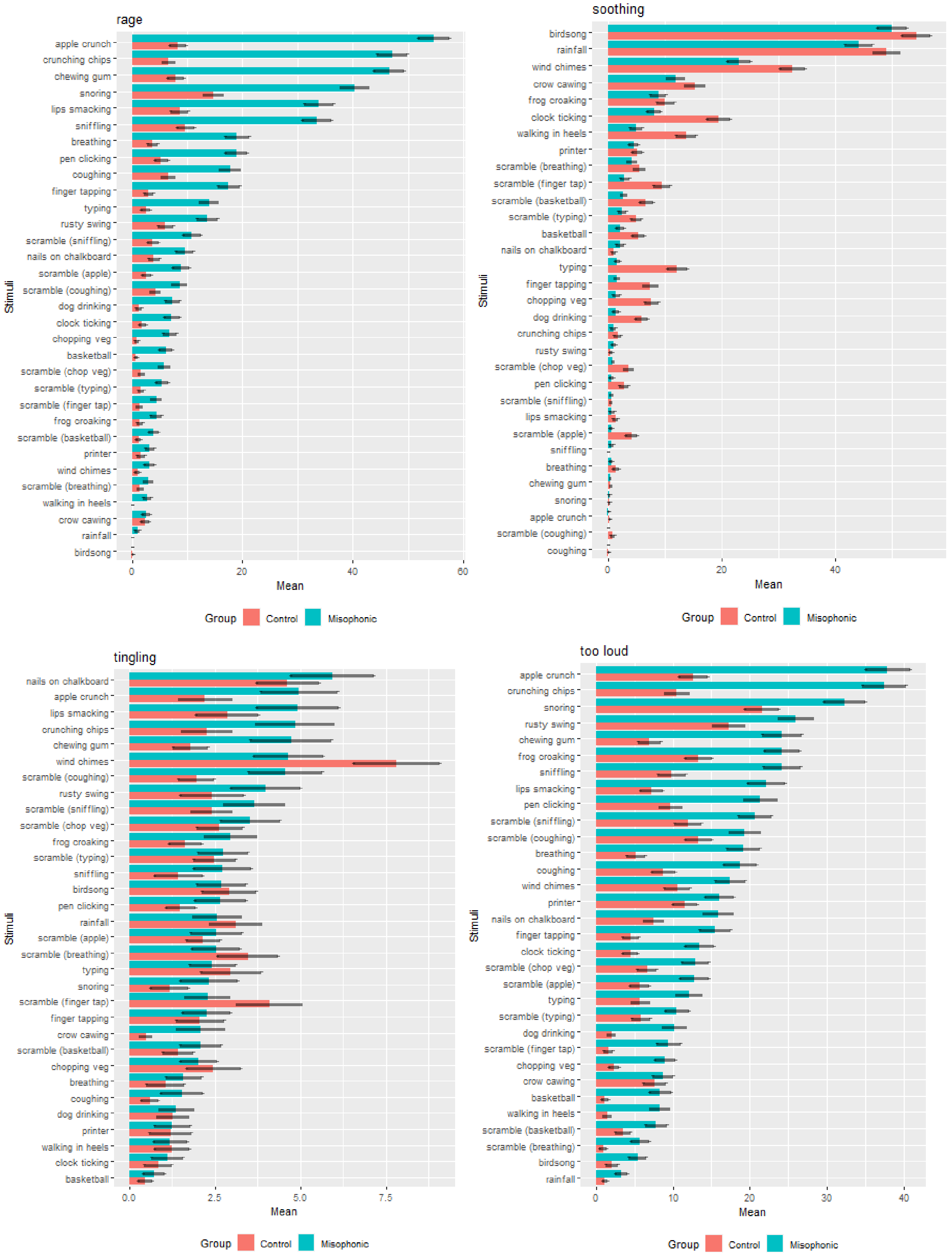

Figure S12. Mean ratings for the two groups (ranked by the mean for the misophonic group) for visual experiences. Error bars: +/- 1 SEM. Related to Figure 4.

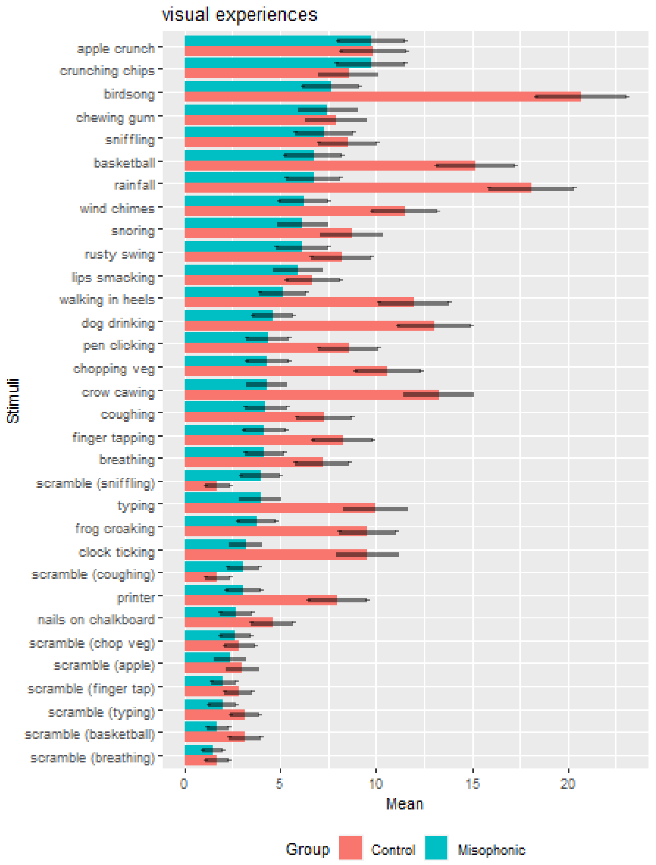

Figure S13: Heatmaps (showing Cohen's d effect sizes) for 32 sounds (y-axis) and 17 responses (x-axis) contrasting moderate misophonia against non-misophonics (top) and contrasting severe against moderate misophonia (bottom). The left panels display all effect sizes. The right panels displays the effect sizes for significant results (p < .05 uncorrected). The distinction between non-misophonics and moderate misophonics is characterized by large differences around typical misophonic triggers, and smaller differences elsewhere. By contrast, the distinction between moderate and severe misophonia is more broadly distributed across a wide range of sounds (not just typical triggers) and additional kinds of responses (e.g. pain, headache) become more prominent. Related to Results (Differential Diagnostic Profiles: Within Misophonics).

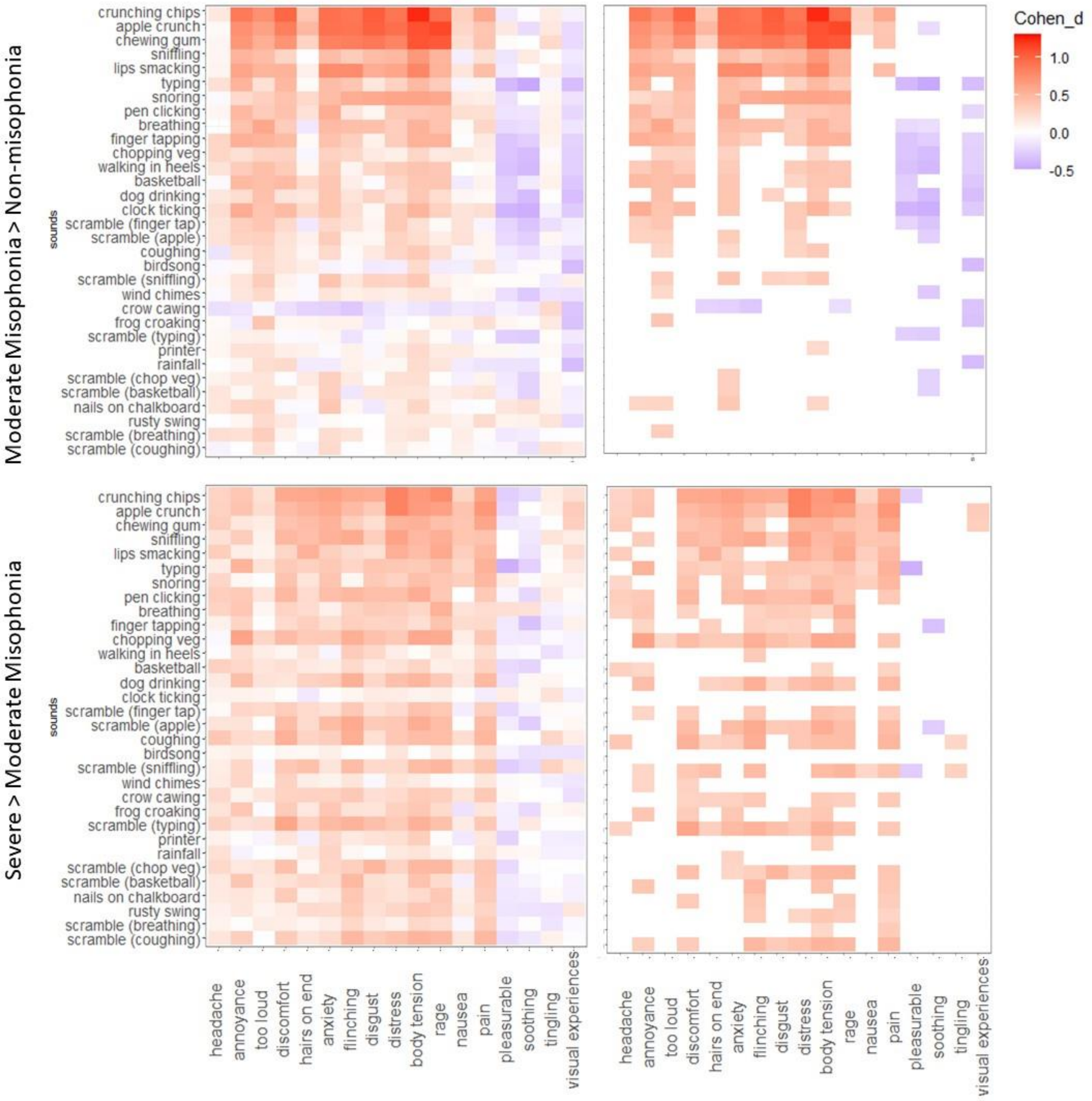

Figure S14: Heatmap (showing Cohen's d effect sizes) for 32 sounds (y-axis) and 17 responses (x-axis) for comparisons within non-misophonics, contrasting high versus low sensory sensitivity on the GSQ. The sounds which best discriminated high v. low GSQ were in descending order: sniffling (AUC=0.635), lips smacking (AUC=0.627), coughing (AUC=0.623), scrambled coughing (AUC=0.622), rusty swing (AUC=0.621), breathing (AUC=0.614), scrambled apple crunch (AUC=0.612), and chopping vegetables (AUC=0.608). Although all eight of these were significantly different from chance, none survived FDR correction for multiple comparisons. Related to Results (Differential Diagnostic Profiles: Within Non-misophonics).

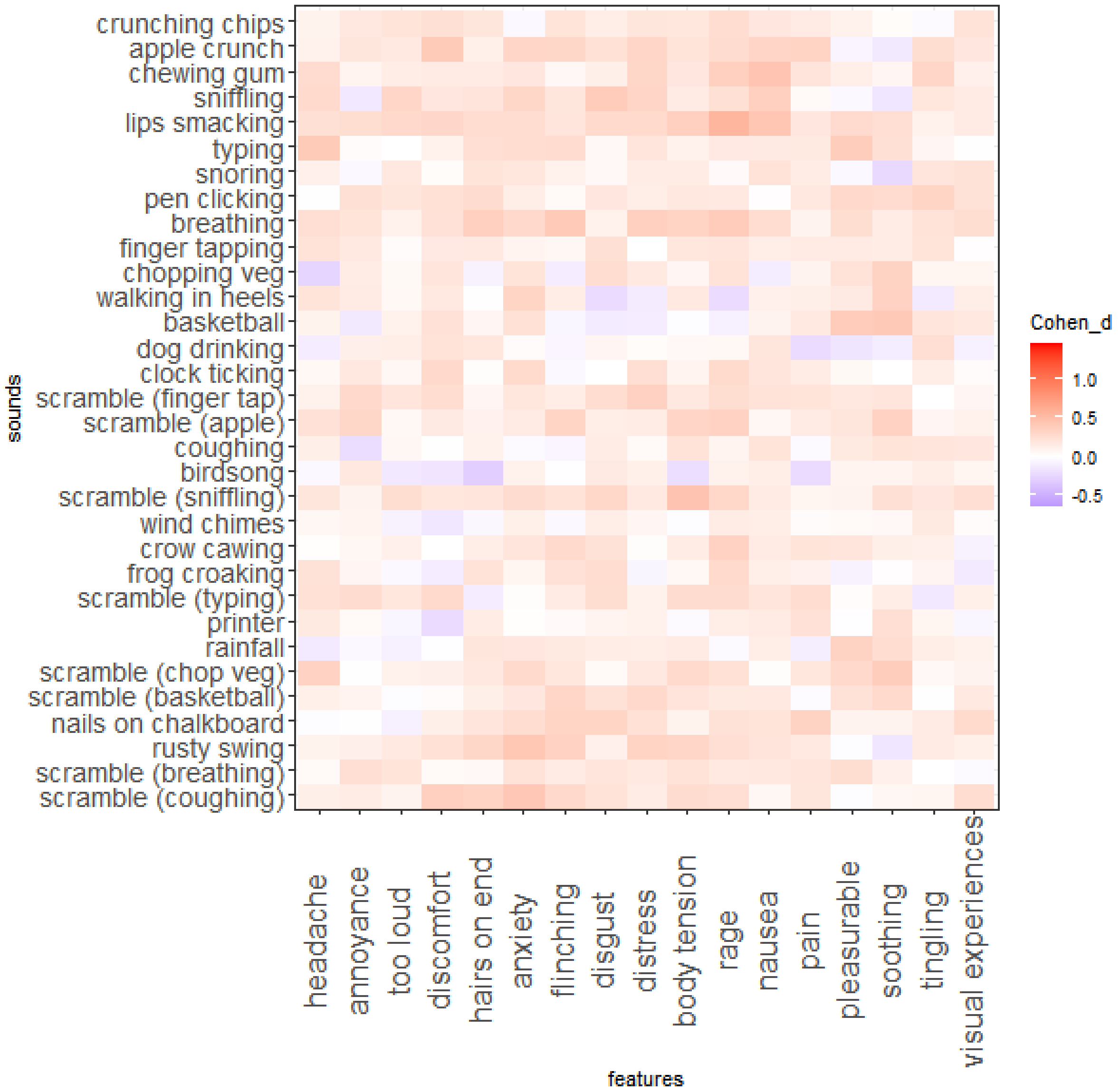

Figure S15: Heatmap (showing Cohen's d effect sizes) for 32 sounds (y-axis) and 17 responses (x-axis) for comparisons within non-misophonics contrasting high versus low interoceptive sensibility on the MAIA. There was little or no discernible trend. Notably, there is no convincing evidence of more somatic responses to sounds in people high on this measure (in fact the most consistent response was higher visual experiences). There was one sound that enabled the groups to be discriminated above chance (wind chimes, AUC=0.650) and it did not survive FDR correction. Related to Results (Differential Diagnostic Profiles: Within Non-misophonics).

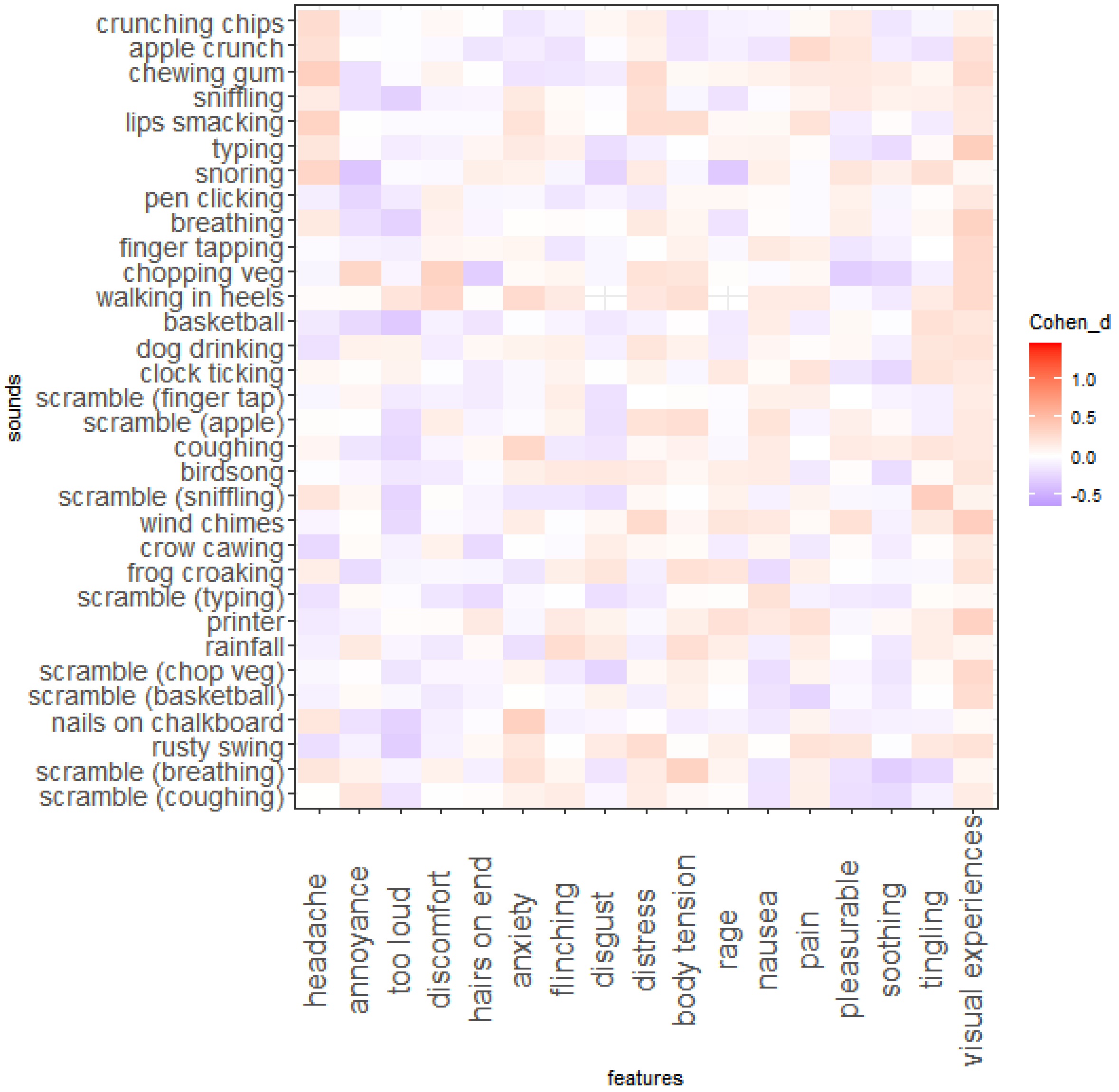

Figure S16: Top – Heatmap showing misophonics versus non-misophonics (taking a cut value of 50.5 on the SMS and excluding all participants with high ASMR) for the eight ASMR trigger sounds. Bottom – the equivalent data for participants with both ASMR and Misophonia relative to participants with neither. Note that for this ‘double diagnosis’ group, the ASMR pattern is dominant. Related to Figure 8.

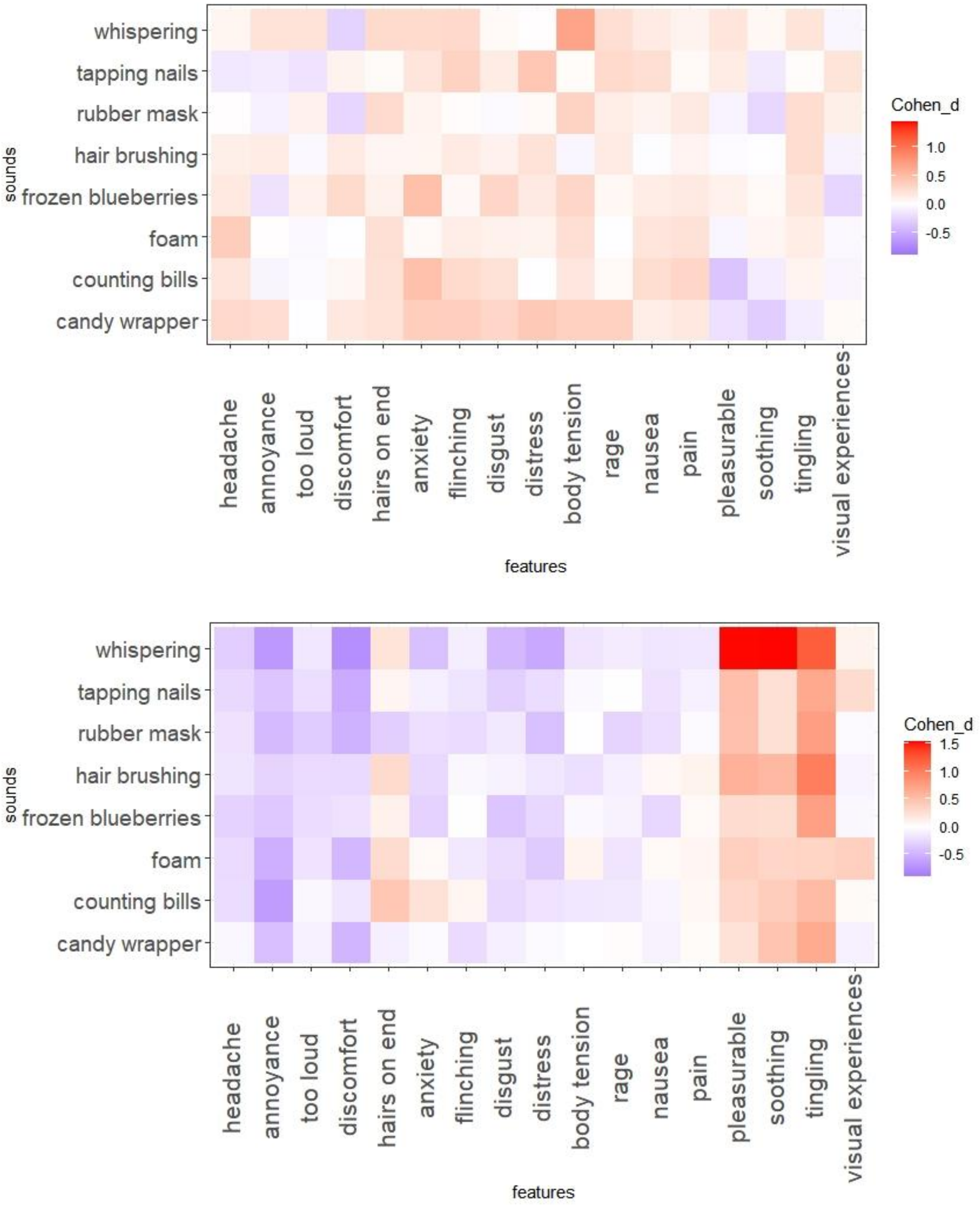

Table S1. Sounds ranked according to which they were most likely to induce ASMR, and their corresponding average start and peak times (N = 24). Fifteen sounds were taken from the website 'freesound' (URL: https://freesound.org). One was taken from YouTube ASMR creator 'xokatie ASMR' (https://youtube.com/xokatieASMR). * = chosen for main study. Related to STAR Methods (Sound stimuli).

Sound	N experiencing ASMR	Average ASMR start-time (seconds)	Average ASMR peak-time (seconds)
*Whispering poems	18	17.52	33.13
*Candy wrapper	18	19.57	34.62
*Foam	15	22.41	32.01
*Hair brushing	15	30.39	46.18
*Counting bills	14	23.40	35.21
*Frozen blueberries	13	27.39	41.59
*Rubber mask	12	16.65	26.44
*Tapping nails	12	16.96	33.23
Turning pages	12	25.62	46.94
Microphone scratching	11	13.71	25.7
Summer night	10	16.35	30.59
Wrapper sounds	10	17.47	30.94
Mouth sounds	8	18.24	25.39
Coins in a bowl	8	24.68	34.42
Typing	8	19.83	39.35
Popcorn	6	38.86	50.65
